# Cryo-EM Reveals How Human Cytoplasmic Dynein Is Auto-inhibited and Activated

**DOI:** 10.1016/j.cell.2017.05.025

**Published:** 2017-06-15

**Authors:** Kai Zhang, Helen E. Foster, Arnaud Rondelet, Samuel E. Lacey, Nadia Bahi-Buisson, Alexander W. Bird, Andrew P. Carter

**Affiliations:** 1 MRC Laboratory of Molecular Biology, Cambridge CB2 0QH, UK; 2 Max Planck Institute of Molecular Physiology, 44227 Dortmund, Germany; 3 Department of Pediatric Neurology, Université Paris Descartes, Imaging Institute, INSERM U781, Paris, France

**Keywords:** motor, dynein, dynactin, cryo-EM, microtubule, phi-particle, auto-inhibition, activation

## Abstract

Cytoplasmic dynein-1 binds dynactin and cargo adaptor proteins to form a transport machine capable of long-distance processive movement along microtubules. However, it is unclear why dynein-1 moves poorly on its own or how it is activated by dynactin. Here, we present a cryoelectron microscopy structure of the complete 1.4-megadalton human dynein-1 complex in an inhibited state known as the phi-particle. We reveal the 3D structure of the cargo binding dynein tail and show how self-dimerization of the motor domains locks them in a conformation with low microtubule affinity. Disrupting motor dimerization with structure-based mutagenesis drives dynein-1 into an open form with higher affinity for both microtubules and dynactin. We find the open form is also inhibited for movement and that dynactin relieves this by reorienting the motor domains to interact correctly with microtubules. Our model explains how dynactin binding to the dynein-1 tail directly stimulates its motor activity.

## Highlights

• Cryo-EM shows human cytoplasmic dynein-1 in its auto-inhibited, phi-particle form
• Phi-particle disruption in vitro and in cells reveals its role in dynein regulation
• There is a transition from phi-particle to open-dynein: both forms are inhibited
• Dynactin binds open-dynein and aligns its motors to activate processive movement

## Graphical Abstract

**Figure:**
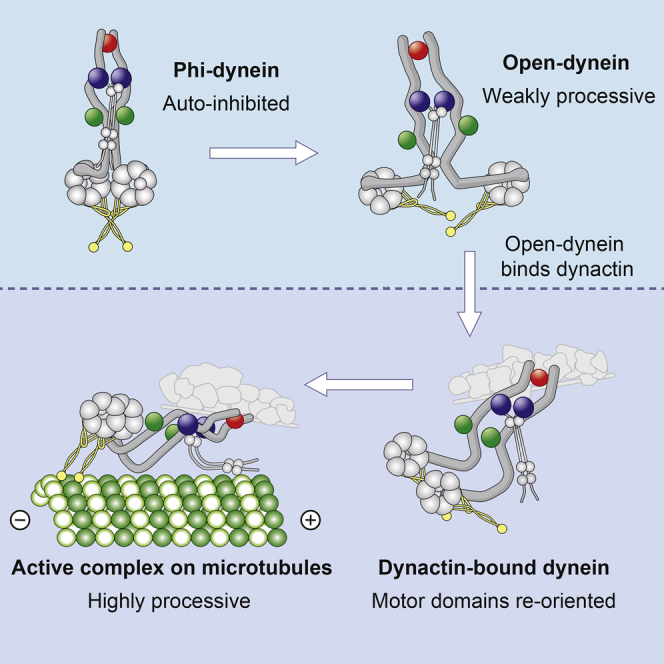

## Teaser

Cryo-EM of human cytoplasmic dynein-1 reveals the mechanism underlying its auto-inhibition and activation.

## Introduction

Cytoplasmic dynein-1 (dynein) associates with dynactin to form an efficient microtubule motor that transports cargo to the minus end of microtubules and organizes the internal components of eukaryotic cells. Disruption of this 2.4-megadalton machine disperses the Golgi network (Burkhardt et al., 1997), blocks transport between organelles (Presley et al., 1997), and leaves viruses stuck at the cell periphery (Döhner et al., 2002). In addition, dynein and dynactin are required during cell division for spindle formation and correct chromosome alignment (Echeverri et al., 1996). Dynein must therefore be carefully regulated to ensure the correct timing and location of motor activation.

In cells, most dynein is diffuse in the cytoplasm, with only a small fraction on microtubules (Splinter et al., 2012). This prevents dynein from inappropriately saturating microtubules or traveling unnecessarily and ensures there is a pool of dynein ready to transport cargos when required. The switch of dynein and dynactin from diffuse to actively transporting cargo is controlled at many levels. It can be driven both in vitro (Mallik et al., 2005) and in vivo (Rai et al., 2013) by clustering motors and influenced by targeting dynein/dynactin to specific post-translationally modified microtubules (McKenney et al., 2016, Nirschl et al., 2016) or the microtubule plus ends (Duellberg et al., 2014, Moughamian et al., 2013). The switch is also controlled at the level of the dynein/dynactin machinery itself. Whereas isolated human dynein is weakly processive in vitro (Trokter et al., 2012), it can be activated to move over long distances (>500 nm) by binding to dynactin and a cargo-specific adaptor protein such as BICD2 (McKenney et al., 2014, Schlager et al., 2014) or Hook3 (McKenney et al., 2014, Olenick et al., 2016, Schroeder and Vale, 2016). This binding stimulates processive movement by increasing the run length, velocity (McKenney et al., 2014, Schlager et al., 2014), and force output (Belyy et al., 2016) of individual motors.

Dynein consists of two motor domains that are responsible for ATP hydrolysis and force production and a tail region that holds them together. It is unclear why dynein is only weakly processive on its own and how it is activated by dynactin and cargo adaptors. There is some evidence that dynein processivity is directly inhibited by the C-terminal ∼300 amino acids of the motor domain (Nicholas et al., 2015). Another theory is that inhibition is due to the tail region folding back to contact the motor domains until cargo binds (Belyy et al., 2016, Markus et al., 2009). A similar inhibition mechanism is used by cytoskeletal motors in the kinesin (Kaan et al., 2011) and myosin families (Hammer and Sellers, 2011). Alternatively, it has been proposed that dynein is auto-inhibited by self-dimerization of its motor domains (Torisawa et al., 2014). This form of dynein is referred to as the phi-particle because of its resemblance to the Greek letter phi (φ) (Amos, 1989). Activation was suggested to result from a shift in the equilibrium of dynein conformations toward an open form in which the motor domains are separated. In support of this, forced separation of isolated dynein motor domains can increase motor activity (Torisawa et al., 2014). However, these studies were performed on artificially dimerized dynein motors lacking the tail region. It is therefore not clear whether the tail contributes to inhibition or what role the phi-particle plays in the context of the whole dynein complex.

In this study, we set out to determine whether the phi-particle contributes to dynein auto-inhibition and how dynein is activated by dynactin. We use cryoelectron microscopy (cryo-EM) to determine the structure of the phi-particle. We show how the motor domains self-dimerize and are locked in a conformation with weak affinity for microtubules. Disrupting the motor dimer by structure-based mutagenesis drives dynein into an open form with increased affinity for microtubules and dynactin. Surprisingly, we discover that the open form of dynein is also inhibited. We use a combination of 2D analysis of EM images and a 3D cryo-EM structure of the whole dynein/dynactin machinery to explain how dynactin overcomes this inhibition and directly reorients the motor domains to make dynein processive. Finally, we show that disrupting the phi-particle in cells causes mis-localization and mitotic defects, supporting a physiological role for the phi-particle in dynein regulation.

## Results

### Structure Determination of the Human Dynein-1 Phi-Particle

**Figure 1:**
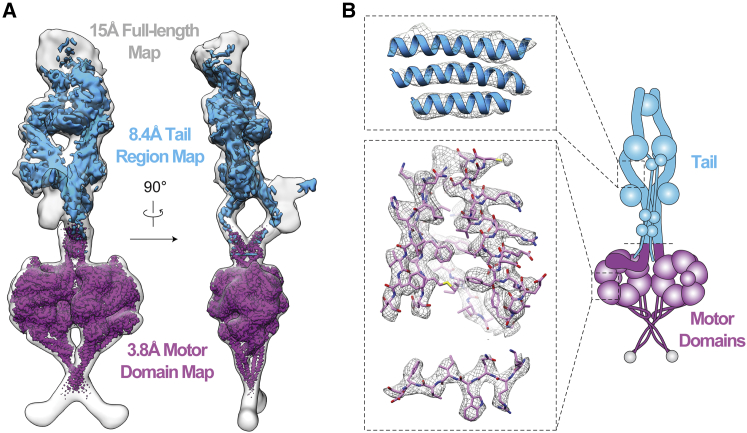
Cryo-EM 3D Reconstruction of Full-Length Human Dynein-1 (A) Overview of the dynein-1 phi-particle. Surface rendering of dynein-1, unsharpened and low-pass filtered to 15 Å (transparent gray) containing the 8.4 Å structure of the tail (cyan, sharpened map) and 3.8 Å structure of the self-dimerized motor domains (purple, sharpened map). (B) Representative electron density of α helices in tail and side chains in the motor domains. Cartoon colored as in (A). See also Figure S1, Table S1, and Movies S1 and S2.

**Figure S1:**
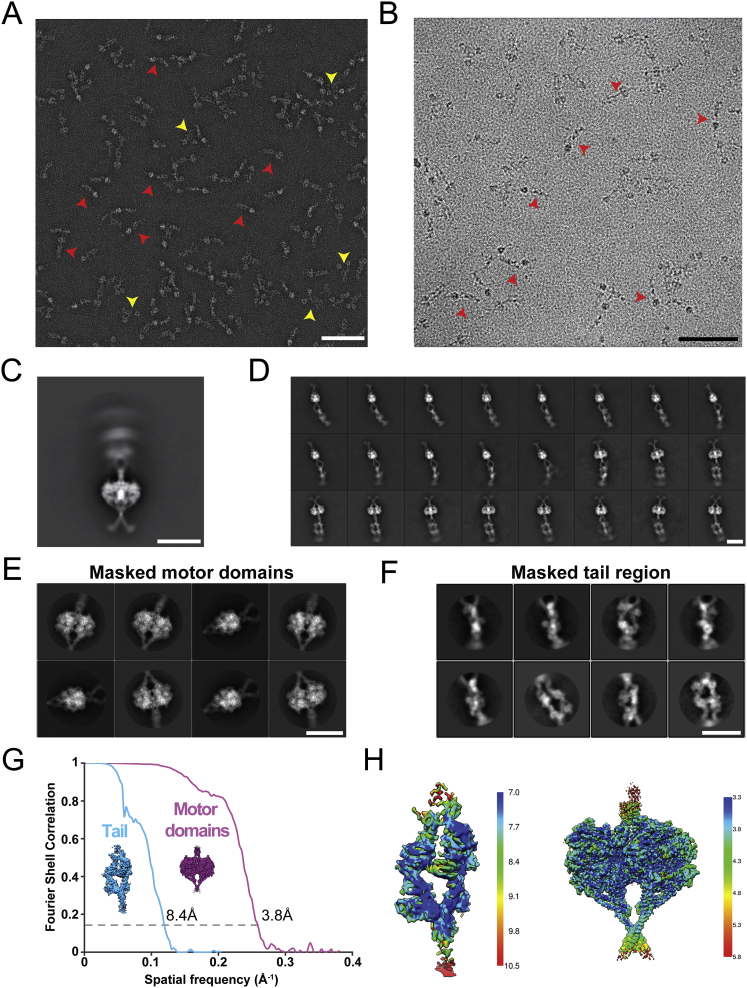
Cryo-EM Image Processing Summary, Related to Figure 1 (A) Negative-stain electron micrograph of the recombinant human dynein-1. Red arrowheads indicate phi-dynein and yellow arrowheads indicates open-dynein. Scale bar 100nm. (B) Example cryoelectron micrograph of recombinant human dynein-1 used for the 3D reconstructions. Red arrowheads show examples of phi-dynein particles. Scale bar 100nm. (C) Initial 2D classification of the cryo-EM data shows that the motor domains align well, but the tail shows significant conformational flexibility with respect to the motor domains and so appears blurred. Scale bar 20nm. (D) Further 2D sub-classification reveals the relative movement between the tail and motor domains, as well as flexibility within the tail region itself. Scale bar 20nm. (E) The 2D class averages of motor domains obtained by reclassification of recentered particles which have been masked to exclude the tail. Scale bar 20nm. (F) The 2D class averages of tail obtained by reclassification of recentered particles which have been masked to exclude the motor domains. Scale bar 20nm. (G) FSC curves of the best resolved classes of the motor domain (purple) and tail (cyan). (H) Local resolution of the two cryo-EM density maps, as plotted by ResMap. Most regions of the tail are resolved to a resolution of ∼7-9Å. The motor domain resolution ranges from ∼3.3Å in the core to ∼4.3Å on the surface.

To understand the role of the phi-particle in dynein auto-inhibition, we solved the structure of full-length human cytoplasmic dynein-1 by cryo-EM (Figure 1A). In initial negative stain EM images, we observed dynein in a mixture of forms; the phi-particle (phi-dynein) and an open form where the motors are separated (open-dynein) (Figure S1A). We used exclusively freshly purified protein produced in insect cells for structure determination. Under these conditions, the proportion of phi-dynein is greater than 85%.

To determine the structure, we collected 714,571 raw dynein particles in vitrified ice (Figure S1B; Table S1). Initial 2D classification revealed the dynein tail was highly flexible relative to the motor domain dimer (Figure S1C). Sub-classification allowed us to visualize the flexibility of the tail (Figure S1D; Movie S1) and to solve a 15 Å resolution structure of the whole phi-particle (Figure 1A). We locally masked either the tail or the motor domain and processed these independently to obtain higher resolution information (Figures S1E and S1F). The main tail class was refined to an 8.4 Å reconstruction (Figures S1G and S1H) that contains clearly defined secondary structure features (Figure 1B). The map of the motor domain dimer reached 3.8 Å resolution (Figures S1G and S1H), allowing us to build a full atomic model (Figure 1B; Table S1). We fit the separate motor domain and tail densities into our 15 Å map of full-length dynein to reveal the architecture of the entire complex (Figure 1A; Movie S2).

### The Architecture of the Highly Flexible Tail Domain

**Figure 2:**
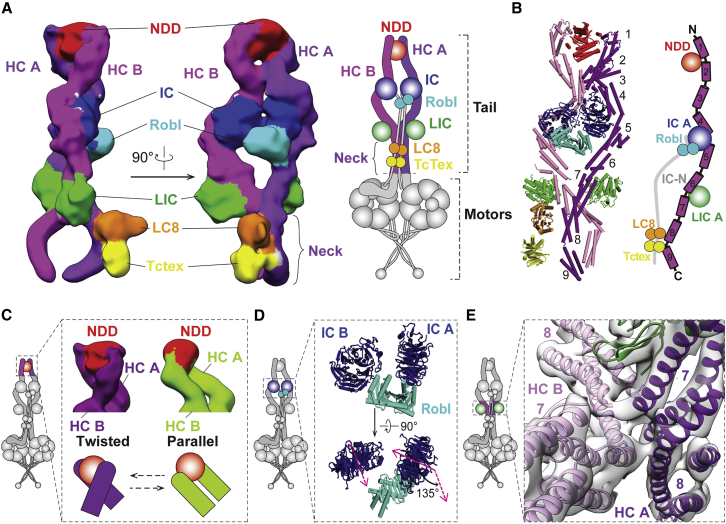
Architecture of the Dynein-1 Tail (A) An unsharpened map of the tail, low-pass filtered to 15 Å. Subunits are colored as in the cartoon. The heavy chain (HC) N-terminal dimerization domain (NDD) holds HC A and HC B together. The HCs bind the intermediate chains (IC) and light intermediate chains (LIC). The light chains Robl, LC8, and Tctex bind to the IC N-terminal extended region (gray in cartoon, not visible in the map). (B) Ribbon and cylinder representation of the tail colored as in (A). Helical bundles 1–9 and accessory chain binding sites are shown with the IC N terminus (IC-N) in cartoon of HC A (right). (C) Unsharpened maps of the tail, low-pass filtered to 15 Å, in either its twisted (dark purple) or parallel (light green) form show the NDD sitting between helical bundles 1 and 2. (D) Robl binds the N-terminal extended region of IC A and IC B holding the ICs at a 135° rotation relative to each other. Robl is closer to IC A than IC B. (E) Contact site between HC helix bundles 7 and 8. HC A, HC B, and LIC A (green) are shown in the 8.7-Å electron density map (transparent gray). See also Figures S2 and S3 and Movies S1, S3, and S4.

**Figure S2:**
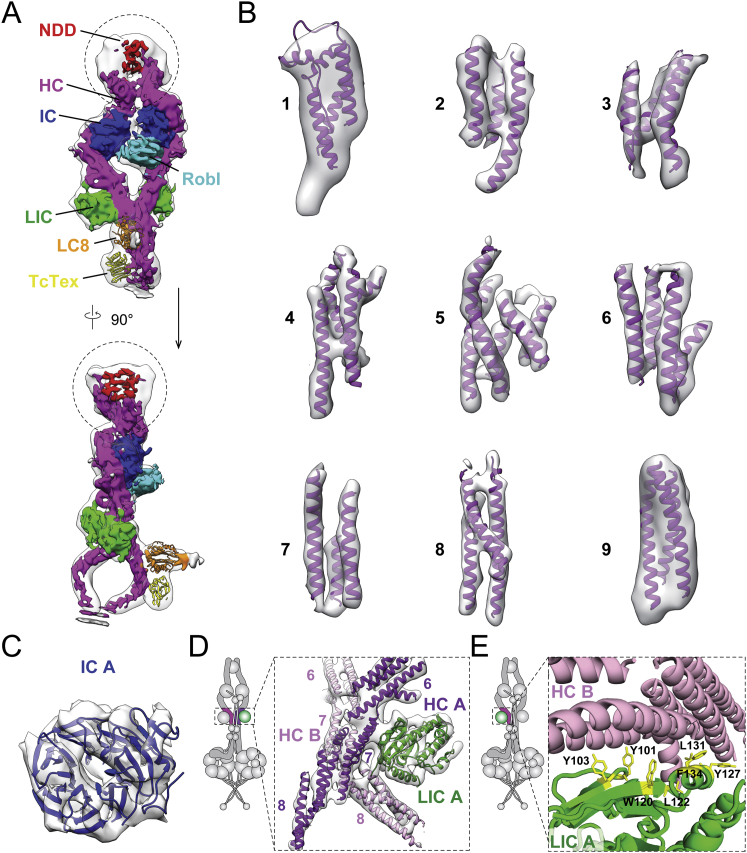
Architecture of the Sharpened 8.4-Å Tail Map, Related to Figure 2 (A) Segmentation and identification of all the subunits in tail as in Figure 2A, but using the sharpened 8.4Å map. The dotted circles indicate the most flexible parts of the map in which the density is over-sharpened. (B) Electron density of the nine HC helical bundles in the tail, containing the fitted (1), built (2-8) or modeled (9) helical bundles in ribbon representation. Density shown for bundles 2-8 is from the sharpened map and for bundles 1 and 9 is from the un-sharpened, low-pass filtered map. We assume all the density shown corresponds to the HC, but do not rule out the possibility some is contributed by elements in ICs and LICs. (C) Electron density map and the fitted homology model of the β-propeller WD40 domain of the IC. (D) The interaction between HC bundles 6 - 7 and the LIC. The electron density map is shown in the surface representation (transparent gray) and is fitted with the helical bundles of HC A (purple) and HC B (pink) and the homology model of human LIC (green), generated from *Chaetomium thermophilum*. (E) Ribbon representation of the contact region between LIC B and HC B. A series of conserved hydrophobic residues of the LIC (yellow, stick representation) are seen to interact with the HC.

The dynein tail is an essential part of the dynein/dynactin transport machine, linking the two motor domains to dynactin and cargo adaptors. Our complete 3D structure of the tail (Figures 2A and S2A) agrees well with the overall architecture proposed in previous 2D negative stain EM (Chowdhury et al., 2015). It is formed of two copies of the dynein heavy chain (HC) each containing a 200-amino-acid N-terminal dimerization domain (NDD) (Urnavicius et al., 2015) followed by nine α-helical bundles (Figures 2B and S2B). Each dynein HC binds to a dynein intermediate chain (IC) and light intermediate chain (LIC). We fit a homology model of the IC C-terminal WD40 domain into density (Figure S2C) that contacts the HC bundles 4 and 5 (Figure 2B). We also docked a homology model of the human LIC into our map (Figure S2D). The LIC binds to the HC bundles 6 and 7 (Figure 2B) using a series of conserved hydrophobic residues near its N terminus, in agreement with previous predictions (Schroeder et al., 2014) (Figure S2E).

**Figure S3:**
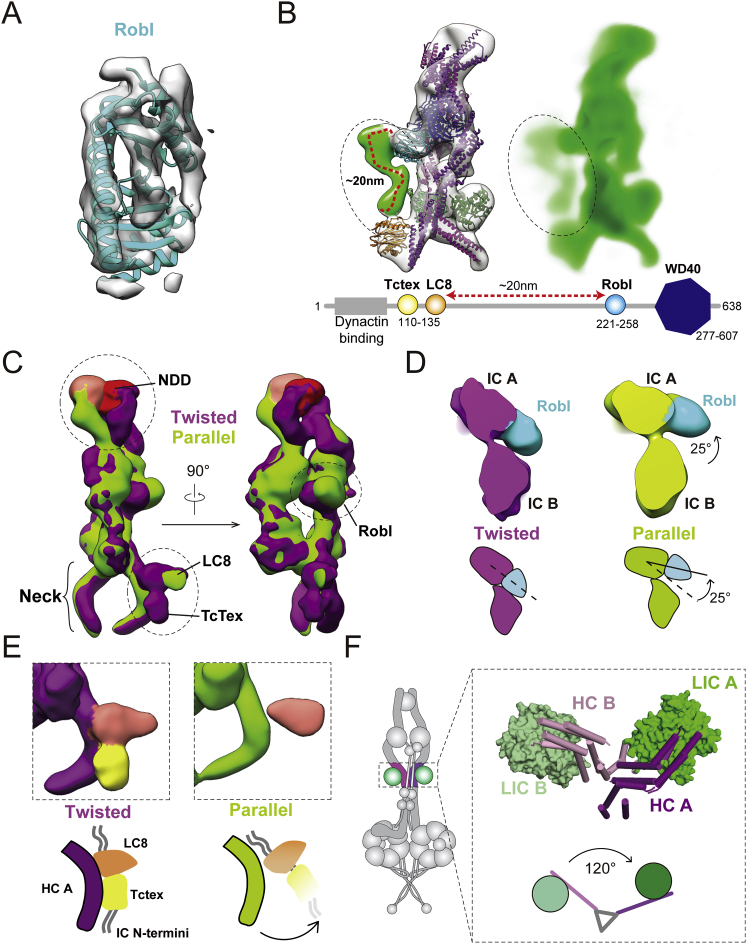
Structural Details of Accessory Chain Binding, Related to Figure 2 (A) Electron density map and fitted Robl solution structure. (B) Electron density map of a typical dynein tail class showing electron density between Robl and LC8 (left). The green region was not assigned to any previously known structure, but its length (∼20nm curve) is compatible with the predicted length of the IC linker region sequence (bottom). A projected electron density representation (right) shows weak electron density in this region (dotted circle), indicating inherent flexibility. (C) A structural comparison of the two major tail conformations – parallel (green) and twisted (purple). Dotted circles correspond to areas with significant structural changes between the two conformations: N-terminal dimerization domain (top left), Robl (right), and LC8/Tctex (bottom left). (D) Close up view of Robl region as in C with cartoon to illustrate 25° rotation. (E) Close up of LC8/Tctex region as in C with cartoon to show probable movement of the LCs. (F) Cylinder and surface representation of HC contact site near the LIC. This region has 120° rotational symmetry.

The dynein tail also contains dimers of three light chains (LCs: Robl/LC7, Tctex, LC8), which bind the extended N terminus of the IC. We see a three-layered density adjacent to the IC WD40 domains, which fits the Robl dimer (Figure S3A) (Song et al., 2005). This density lies on one side of the dynein tail (Figure 2A). On this same face, we see additional density docked to the neck region of HC A (Figure 2A). We tentatively assign this to the structure of LC8 and Tctex dimers (Williams et al., 2007) on account of weak density linking it to Robl. This curved linkage is ∼20 nm long, consistent with the length of the N-terminal IC polypeptide known to connect Robl and LC8 (Figure S3B). Previous negative stain EM studies on open-dynein show the LCs trailing away from the tail (Chowdhury et al., 2015). Therefore, the LC8/Tctex interaction with the HC may be specific to phi-dynein.

The HCs in the phi-particle tail are held together at three main sites. The first contact site is the NDD, which lies between helical bundles 1 and 2 (Figure 2B). This part of the tail is dynamic and adopts multiple conformations. Extensive sub-classification revealed a “twisted” form that is dominant, a less populated “parallel” state (Figures 2C and S3C; Movie S3), and a variety of intermediate states. Of these, the parallel state is most similar to the conformation of this region when bound to dynactin (Urnavicius et al., 2015). Interestingly, the transition from twisted to parallel correlates with small shifts in the relative position of the ICs and larger changes in the position of the LCs: Robl rotates through 25° (Figure S3D), and the LC8/Tctex density moves away from the HC neck (Figure S3E). This suggests that conformational changes in the very N terminus of the HCs can be transmitted over long distances in the tail. The second contact site results from Robl binding to the IC extended N terminus and holding the IC WD40 domains together. Robl is much closer to IC A (Figures 2D and S3D), suggesting it is constrained by connections to the IC WD40 domains. The binding of Robl to both IC N termini is likely to be responsible for orienting the ICs, which are related to each other by a 135° rotation about the long axis of the tail (Figure 2D). The third HC contact site, which has not been described previously, is close to the LICs and involves a direct interaction between HC helical bundles 7 and 8 (Figures 2E and S3F; Movie S4). In a previous negative stain EM study of open-dynein (Chowdhury et al., 2015), the first and second contact sites were identified but the distance between the LICs suggests that the third contact site was not present. This suggests that the LIC-proximal contact site acts to stabilize motor-domain self-dimerization in phi-dynein.

### Self-Dimerization Holds the Dynein Motors in a Weak Microtubule Binding State

**Figure 3:**
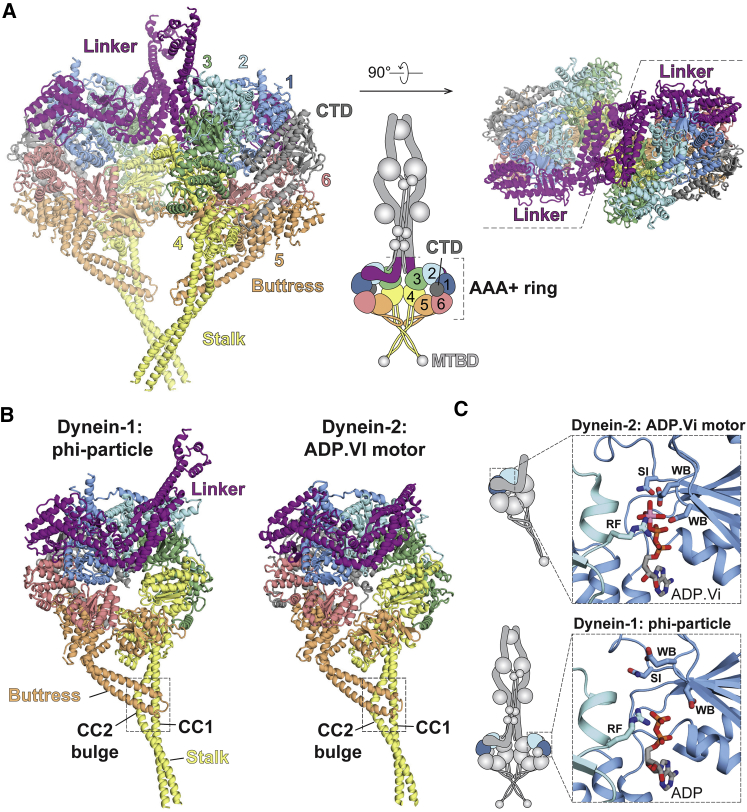
Motor Dimerization Locks Phi-Dynein into a Weak Microtubule Binding State (A) The dimeric motor domains in the phi-particle from the side (left) and top (right), showing the linker (purple), ring of six AAA^+^ domains (colored as in cartoon), stalk (yellow), buttress (orange), and C-terminal domain (CTD, in gray). MTBD, microtubule binding domain. (B) The motor domains from the phi-particle and the crystal structure of cytoplasmic dynein-2 bound to ADP.vanadate (ADP.Vi, PDB: 4RH7). Coiled-coil helices 1 and 2 (CC1 and CC2) control microtubule affinity. Both motors display a bent linker and stalks that have low microtubule affinity due to the bulge in CC2. (C) AAA1 nucleotide binding sites are similar in ADP.Vi-bound dynein-2 and the ADP-bound dynein-1 phi-particle. The main catalytic residues are labeled: WB, Walker B; SI, Sensor-1; RF, Arginine finger. See also Figure S4.

A dynein motor domain consists of a linker that is a continuation of the HC neck, a ring of six AAA^+^ domains, and a coiled-coil stalk that binds to microtubules at its tip. In the phi-dynein structure, the linkers of the two motor domains contact each other in the center of the dimer (Figure 3A). The C-terminal domain (CTD) of the motor, which was previously hypothesized to be part of the dimerization interface (Torisawa et al., 2014), is on the outside of the motor dimer (Figure 3A). In the phi-particle, the stalks are diametrically opposed to the linker N termini (Figure 3A) and therefore also the tail (Figure 1A). The conformation of the motor domain in phi-dynein is similar to the crystal structure of a related dynein (cytoplasmic dynein-2) trapped in the presence of the ATP hydrolysis transition state analog, ADP.vanadate (ADP.Vi) (Schmidt et al., 2015) (Figure 3B). The ADP.Vi structure represents the conformation of dynein just before it binds to microtubules and undergoes a powerstroke.

**Figure S4:**
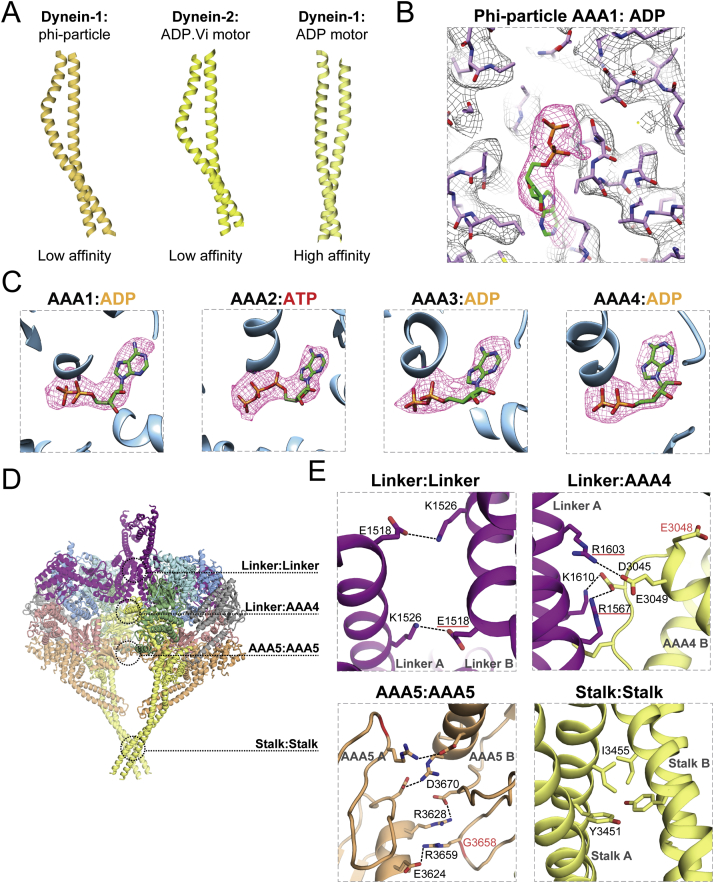
Structural Details of the Phi-Particle Motor Domains, Related to Figure 3 (A) Comparison of coiled-coil stalks in our dynein-1 phi-particle structure (left) and crystal structures of the low microtubule affinity, ADP.Vi bound dynein-2 motor (middle, PDB: 4RH7) and the high microtubule affinity, ADP bound dynein-1 motor (right, PDB: 3VKG). (B) Stick representation of the phi-particle AAA1 active site showing the ADP electron density (red mesh) and surrounding residues (transparent mesh). (C) The electron density maps (red mesh) suggests that nucleotide binding sites AAA1, AAA3 and AAA4 contain ADP, while AAA2 contains an ATP. (D) Positions of the four main contact sites located at the interface of the motor domain dimer. (E) Enlarged views of each contact site. Electrostatic interactions between residues are marked with black dotted lines. Residues with mutations associated with human neuropathies are underlined in red (at an interface residue) or shown in red text (next to an interface residue).

The microtubule affinity of dynein is controlled by a sliding of the helices in the stalk coiled coil (Schmidt et al., 2015). Similar to the ADP.Vi structure, our motor domains have their stalks in a low-affinity conformation. In both structures, the stalk helix CC2 forms a bulge near to where it binds to the buttress (Figure 3B). This is in contrast to the crystal structure of dynein-1 in the high-affinity, ADP-bound state (Kon et al., 2012) where the stalk helices form a straight coiled coil (Figure S4A). Previous work proposed that the weak affinity of phi-dynein for microtubules is caused by crossing of the stalks, which prevents the two microtubule binding domains (MTBDs) from binding simultaneously (Torisawa et al., 2014). Here, we show dimerization has a more fundamental effect on microtubule affinity by locking the motors into a weak affinity state.

The AAA1 nucleotide binding site is the main site of ATP hydrolysis in dynein. In the ADP.Vi structure it is closed around the nucleotide with many of its key catalytic residues clustered around the vanadate group, which occupies the position of the ATP γ-phosphate (Figure 3C). Our structure was solved in the presence of ATP, but the density suggests only an ADP is bound in AAA1 (Figures S4B and S4C). Surprisingly, the AAA1 site in phi-dynein is tightly closed, similar to the ADP.Vi structure (Figure 3C). It is different from the more open conformation expected for dynein with ADP in AAA1 (Kon et al., 2012). The fact that the active site is closed in the presence of ADP suggests the weak affinity of the motor domains in the phi-particle is stabilized by dimerization rather than by the nucleotide state of AAA1.

### Disrupting Motor Self-Dimerization Increases the Affinity of Dynein for Microtubules and Dynactin

To understand how the phi-particle affects the affinity of dynein for microtubules, we asked whether disrupting motor self-dimerization would alter binding. The self-dimerization interface consists mainly of charged residues that cluster at four sites (Figures S4D and S4E). The two linker domains interact through salt bridges between residues E1518 and K1526. The linker contacts AAA4 on the opposing motor through the positively charged linker residues R1567, R1603, and K1610 and the negatively charged AAA4 residues D3045 and E3049. The AAA5 domains interact through an electrostatic network involving residues R3628, R3659, E3624, and D3670. Finally, the stalks interact at their crossover point via hydrophobic residues Y3451 and I3455.

**Figure 4:**
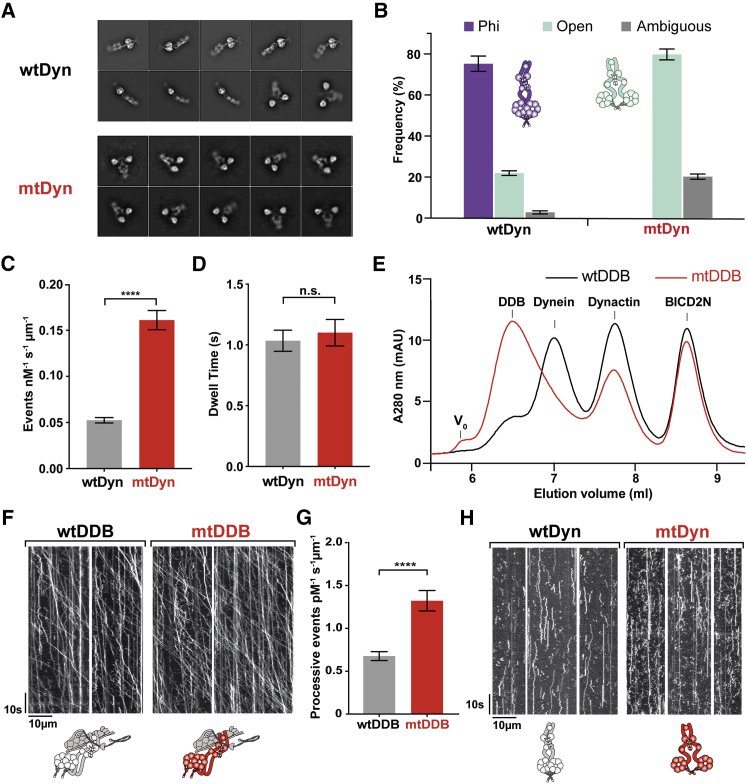
Disruption of Motor Self-Dimerization Increases Microtubule and Dynactin Affinity (A) Representative 2D class averages of negative stain EM images of wild-type dynein (wtDyn) and dynein with interface mutations K1610E and R1567E (mtDyn). wtDyn predominantly adopts the phi-particle form, whereas mtDyn classes are in the open form. (B) Quantification of proportion of phi- and open-dynein in wtDyn and mtDyn. The phi-particle is disrupted in mtDyn. Mean ± SEM is shown. (C) The microtubule association rate of mtDyn is significantly higher than wtDyn. Mean number of events per micrometer microtubule per nM dynein per second is shown ± SEM. ^∗∗∗∗^p < 0.0001 in an unpaired, two-tailed t test. n = 42 microtubules (wtDyn), n = 29 microtubules (mtDyn), three independent experiments. (D) The average length of time spent bound to microtubules (dwell time) for isolated wtDyn and mtDyn is not significantly different. Average dwell was calculated for two-phase exponential decay fits of six repeats for each condition (±SEM). n.s. in an unpaired, two-tailed t test, n = 6. (E) Size exclusion chromatography shows that the mtDyn forms greater amounts of Dynein/Dynactin/BICD2N (DDB) complex than wtDyn. The elution volume of each component and the void volume (V_0_) is indicated. (F) Kymographs of TMR labeled wtDyn or mtDyn mixed with dynactin and BICD2N to form wtDDB or mtDDB show that both complexes can move processively, over long distances on microtubules. (G) mtDDB shows twice the number of processive events that wtDDB. Mean number of processive events (±SEM) are shown per micrometer microtubule per pM dynein. ^∗∗∗∗^p < 0.0001 in an unpaired, two-tailed t test when n = 31 microtubules (wtDDB and mtDDB). (H) Kymographs show that neither wtDyn or mtDyn move processively on microtubules in the absence of dynactin and BICD2N. This suggests both phi- and open-dynein are auto-inhibited. See also Figure S5.

**Figure S5:**
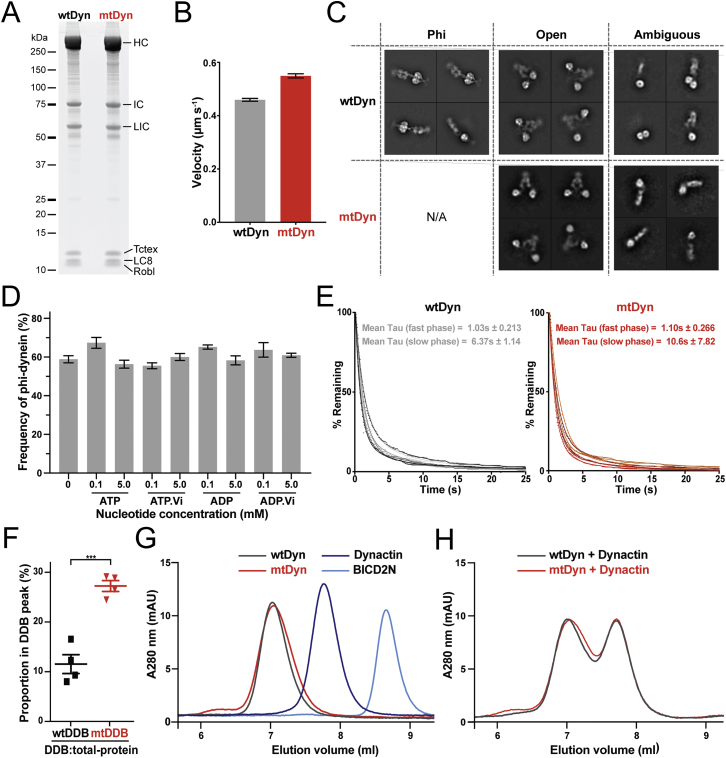
Characterization of Phi-Interface Mutated Dynein, Related to Figure 4 (A) SDS-PAGE of wtDyn and mtDyn samples after SYPRO-Ruby staining show that they have similar subunit stoichiometry. (B) wtDyn and mtDyn motor velocities measured in microtubule gliding assays. Dynein was adhered non-specifically to the glass slide, with the velocity of free microtubules across the field of view determined. wtDyn n = 260 microtubules, mtDyn n = 346. Data shows mean ± SEM. (C) Representative 2D classes from negative stain images of wtDyn or mtDyn identified as being in a phi- or open- or ambiguous conformation, as used for quantification. For mtDyn, no obvious phi-particle classes were observed. Ambiguous classes are those that could not be assigned as phi- or open-dynein. (D) The frequency of phi-dynein observed after 2D classification of wtDyn incubated in the presence of indicated nucleotides. The 0 mM nucleotide sample was obtained by omitting nucleotides from SEC performed in the final step of purification. Contrary to previous reports (Torisawa et al., 2014), we did not observe a significant difference in the proportion of phi-particle in these preparations. Mean ± SEM is shown. (E) Dwell times of isolated wtDyn or mtDyn binding to microtubules were calculated by fitting a two-phase exponential decay model to each independent experiment. Data was plotted as a histogram of the percentage of particles remaining attached to the microtubule for a given amount of time after binding (Bin size = 0.125 s). The average dwell time was taken to be the average time constant of the fast phase (±SD, n > 300 events for each repeat, 6 repeats). The fast phase accounts for ∼80% of the fit. In an unpaired Student’s t test, neither the slow or fast phase fits were significantly different. (F) Analytical SEC has been repeated with 3 different preparations of wtDyn, mtDyn, Dynactin and BICD2N. The proportion of protein in the DDB complex peak relative to the total protein amount was calculated using an area under the curve analysis in GraphPad Prism. On average, wtDDB had 11.5 ± 2% protein in the DDB peak whereas mtDDB had 27.3 ± 1% (Mean ± SEM). ^∗∗∗^p < 0.001 in an unpaired, two-tailed t test when n = 4 for each. (G) Size exclusion chromatography (SEC) shows that the mtDyn and wtDyn elute at similar volumes. Individual traces of dynactin and BICD2N are also shown. The identity of each component was confirmed by SDS-PAGE. (H) SEC of mtDyn or wtDyn mixed with dynactin show that complex formation only occurs in the presence of BICD2N.

To disrupt this interface, we made charge reversal mutations at the linker-AAA4 interaction (K1610E and R1567E) in full-length recombinant dynein (Figure S5A). These mutations do not impair motor velocity in an in vitro microtubule gliding assay (Figure S5B), so we verified their effect on dimerization using negative stain EM and 2D classification (Figure 4A). In wild-type dynein (wtDyn), we observed 22% (±1% SEM) of molecules in the open form and 75% (±4% SEM) in the phi-particle (Figure 4B). The remainder of the particles were difficult to assign to either class (Figure S5C). The phi-particle frequency was independent of added nucleotide (Figure S5D). In interface-mutated dynein (mtDyn), the proportion of open-dynein was 80% (±3% SEM), and no phi-dynein classes were observed (Figure 4B). This suggests that our mutagenesis successfully disrupts the self-dimerization interface and significantly increases the proportion of open-dynein.

To determine the effect of increasing the proportion of open-dynein, we tested the affinity of wtDyn and mtDyn for microtubules. We analyzed the binding of fluorescently labeled dynein to microtubules in a single-molecule total internal reflection fluorescence (TIRF) assay. We observed a 3-fold higher microtubule binding rate for mtDyn than for wtDyn (Figure 4C), which suggests that increasing the proportion of open-dynein increases microtubule association. This is consistent with our structural evidence that phi-dynein motors are locked in a weak affinity state (Figure 3B). The duration of binding events (dwell time) was not significantly different between mtDyn and wtDyn (Figures 4D and S5E) showing interface mutations do not affect the release of dynein from microtubules. We used the binding and dwell time data to estimate K_d_ values, which are 16.4 μM (±4.6 SEM) for wtDyn and 5.22 μM (±2.0 SEM) for mtDyn.

We investigated whether shifting from phi- to open-dynein also influences dynactin and cargo adaptor binding. We incubated the same concentrations of mtDyn or wtDyn with dynactin and an N-terminal fragment of the cargo adaptor BICD2 (BICD2N) and determined the amount of dynein/dynactin/BICD2N (DDB) complex formed by size exclusion chromatography (Schlager et al., 2014). The DDB peak is significantly higher for mtDyn than wtDyn (Figures 4E and S5F), which suggests open-dynein has increased binding to dynactin and BICD2N. Control experiments show that mtDyn has an identical elution profile to wtDyn and that in both cases interaction with dynactin occurs only in the presence of BICD2N (Figures S5G and S5H).

### Disrupting the Phi-Particle Reveals an Additional Level of Dynein Auto-inhibition

Previous work proposed that disrupting phi-dynein would activate the ability of isolated dynein to undergo long-distance movement (Torisawa et al., 2014). To test this hypothesis, we used a TIRF-based motility assay to determine the ability of fluorescently labeled single wtDyn and mtDyn motors to move along microtubules. In the presence of dynactin and BICD2N, both wtDyn and mtDyn showed long movements along microtubules (Figure 4F). This demonstrates that mtDyn is capable of long-range, processive movement when activated. Quantification showed that mtDyn had double the number of processive DDB complexes compared to wtDyn (Figure 4G), consistent with its higher affinity for dynactin and microtubules described above. Surprisingly, in contrast to the original hypothesis, mtDyn was not able to move over long distances in the absence of dynactin and BICD2N. Instead, it displayed diffusive or static movements similar to that observed for wtDyn (Figure 4H). This suggests that, in addition to inhibition by phi-particle formation, isolated open-dyneins are also inhibited from performing long-range movement.

### Dynactin Binding to the Dynein Tail Induces Significant Changes in Motor Orientation

**Figure 5:**
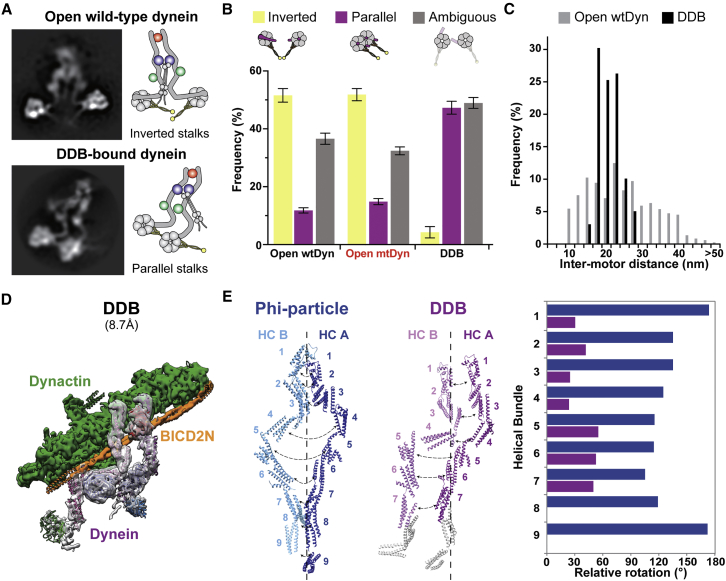
Dynactin Binding Reorients Dynein Motor Domains (A) 2D classification and local refinement of motor domains resolves their orientation. Representative composite images of isolated, open-dynein in negative stain EM and dynein in DDB in cryo-EM (after subtraction of dynactin density). In the dominant open-dynein conformation, the stalks point toward each other (inverted). In DDB they are parallel. Positions of the NDDs (red), ICs (dark blue), and LICs (green) are indicated in each cartoon. (B) Quantification of the proportion of dynein particles with their motor domains inverted, parallel, or in an ambiguous orientation after focused classification. Mean ± SEM is shown. (C) The distribution of distances between motor domains is greater in open-dynein than DDB. Bin size, 2.5 nm. (D) 8.7 Å cryo-EM structure of DDB. Each component is labeled. The dynein motor domains are too flexible to be resolved. (E) Relative rotation between individual helical bundles in each HC in the phi-particle and DDB. Rotation is measured around the long axis of the tail. The HCs of dynein in DDB lie more parallel than in the phi-particle. See also Figure S6 and Movies S5 and S7.

**Figure S6:**
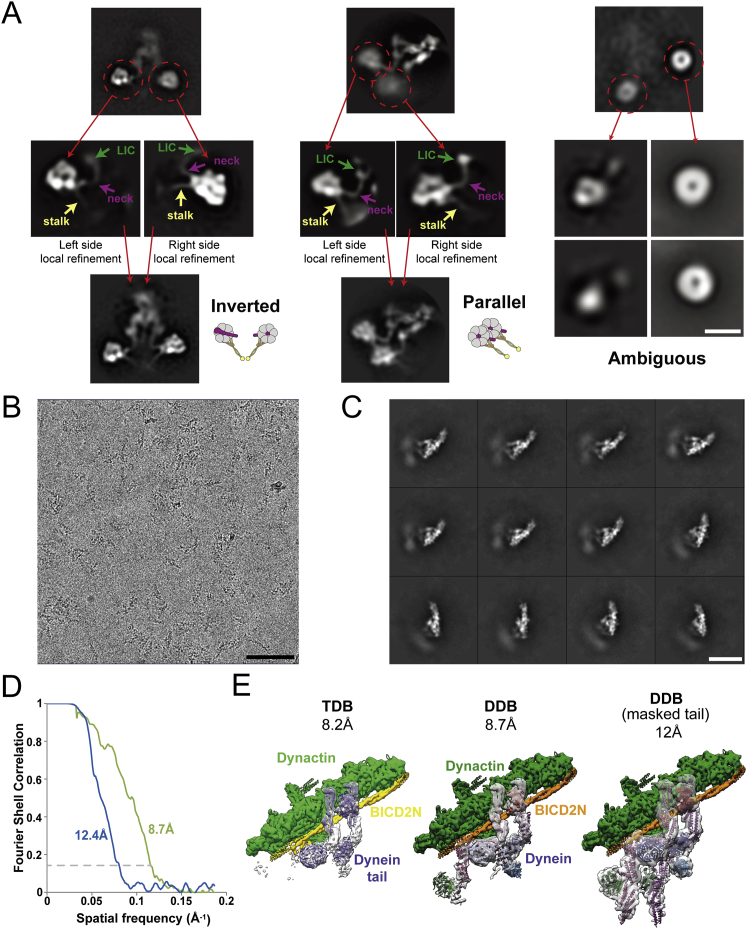
2D and 3D Analysis of Dynein When Bound to Dynactin, Related to Figure 5 (A) Local classification and refinement of each motor domain allowed us to determine the location of stalk, neck and LIC near the motor. We can therefore show that open-dynein (left) predominantly has inverted stalks while DDB-bound dynein has parallel stalks (middle). Examples of ambiguous orientation are also shown (right). Composite images generated in this process are shown below. Scale bar 20nm. (B) Representative cryo electron micrograph of the DDB complex used for the 3D reconstruction. Scale bar 100nm. (C) 2D classification of DDB complexes shows that the tail region near dynactin is stable compared to the motor domains which are more flexible and appear blurred. Scale bar 40nm. (D) The FSC curve of the 8.7Å 3D reconstruction of DDB and the 12.4Å structure of the masked tail from DDB. (E) Comparison of our DDB structures (middle and right) to the previous TDB structure (left) (EMDB: 2861). These structures are similar overall but the DDB structure (middle) contains more electron density in the HC and LIC region. The focused classification structure (right) allows us to build and almost complete model of the tail. In this case, 2D and 3D classification took place on particles that had the dynactin density subtracted. Dynactin and BICD2N are shown as in the DDB (middle) structure for reference.

To understand how open-dynein is inhibited and why dynactin/BICD2N binding activates long-distance movement, we examined the structural differences between the open and DDB-bound forms of dynein. We collected negative stain data of isolated wtDyn or mtDyn and performed extensive 2D sub-classification of their spontaneously open populations. We also obtained 2D class averages of cryo-EM images of the DDB complex after computational subtraction of the dynactin density. The dominant views in these raw 2D classes suggest that the motor domains point toward each other in open-dynein but point in the same direction in DDB-bound dynein (Movies S5 and S6). To resolve these orientations, we performed local classification and refinement of each motor domain in the raw 2D classes (Figure S6A). These were combined to generate composite 2D images in a similar way as previously described for open-dynein (Chowdhury et al., 2015) and allowed us to assign the stalk orientation in each class (Figure 5A).

Quantification showed that, in open wtDyn, 52% (±2% SEM) of particles have stalks that point toward each other (inverted), whereas only 15% (±1% SEM) have stalks that are pointing in the same direction (parallel) (Figures 5A and 5B). The stalk orientation in the remaining particles is ambiguous (Figure S6A). Similar ratios are observed for open mtDyn (Figure 5B). In contrast, the stalk orientation in DDB-bound dynein is only 4% (±2% SEM) inverted, while 47% (±2% SEM) are parallel (Figures 5A and 5B). In addition to changing the orientation of the motor domains, dynactin binding also appears to constrain them. The distance between motor domains in open-dynein ranges from 10 to 45 nm, whereas in the DDB complex the distance is more tightly clustered between 15 and 25 nm (Figure 5C). Therefore, dynactin binding appears to shift the equilibrium of dynein conformations such that the motor domains have reduced flexibility and are more parallel. This matches the orientation observed when dynein is bound to microtubules (Chowdhury et al., 2015, Imai et al., 2015), suggesting dynactin pre-aligns both motor domains for microtubule binding.

We wanted to understand how dynactin binding to the tail influences the orientation of the HC and consequently the motor domains. We therefore determined an 8.7 Å 3D structure of the full DDB complex and a 12.4 Å structure of the locally masked dynein tail when bound to dynactin and BICD2N (Figures S6B–S6D). This shows that the full-length dynein binds to the dynactin filament and BICD2N in a similar way to the isolated dynein tail (Figure S6E) (Urnavicius et al., 2015). The DDB structure, however, contains additional density corresponding to more of the HC between the IC and the neck region (Figure 5D). We fit HC helical bundles, ICs, LICs, and Robl into the DDB density using both 8.7 Å and 12.4 Å maps (Figure S6E).

We then compared the structure of the tail in the phi-particle to that in the DDB complex as these structures represent the start and end point of the dynein activation process. The major change between the phi-particle and DDB structures is a large twist of the whole HC B along its long axis, relative to HC A (Movie S7). The ICs and LICs are tightly bound to the HCs and so move in a concerted fashion with them. We quantified the changes in the HC orientations by measuring the relative rotation between individual helical bundles in HC A and HC B in both phi-dynein and the DDB structure (Figure 5E). The rotation about the long axis of the tail ranges from 100° to 180° in phi-dynein, and so the HCs are related by symmetry of between 2- and 3-fold. In contrast, the rotation between helical bundles in the DDB structure ranges from 20° to 60°. Therefore, the HCs and their associated ICs and LICs are closer to parallel in the DDB (Movie S7). Taken together with our 2D EM data (Figures 5A and 5B), this suggests that dynactin binding stabilizes a parallel orientation of the tail that directly leads to a parallel orientation of the motor domains.

### Mutation of the Self-Dimerization Interface Affects Dynein Localization and Causes Mitotic Defects in Cells

**Figure 6:**
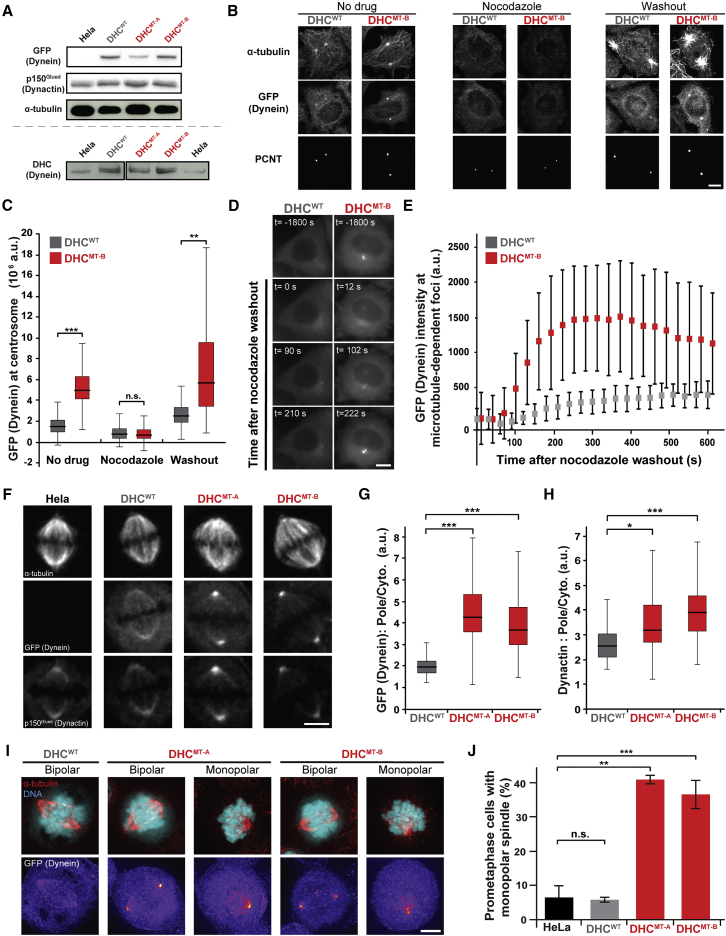
Phi-Particle Disruption Changes Dynein Localization and Causes Mitotic Defects in HeLa Cells (A) Western blots of lysates from HeLa cells and clonal HeLa cell lines stably expressing dynein BAC transgenes. The DHC^WT^ cell line expresses a wild-type GFP-dynein HC (GFP-DHC) transgene. DHC^MT-A^/DHC^MT-B^ are two independent cell lines expressing mutant GFP-DHC (R1567E and K1610E). Top: Blots against GFP, p150^Glued^, and α-tubulin. Expression levels of GFP (dynein) are similar in DHC^WT^ and DHC^MT-B^. Bottom: Blots against DHC. Expression of GFP-DHC (upper band) is similar to or lower than endogenous DHC (lower band) in transgene cell lines. GFP-DHC (upper band) is absent in HeLa lysates. (B) Immunofluorescence with antibodies against α-tubulin, GFP, and pericentrin (PCNT) on DHC^WT^ and DHC^MT-B^ cell lines. G2-synchronized cells were fixed either before nocodazole treatment (No drug), after 30 min with 3.3 μM nocodazole or after washout of the nocodazole for 3 min. Scale bar, 10 μm. (C) Quantification of GFP intensity around individual centrosomes in cells from (B). DHC^MT-B^ show GFP (Dynein) enrichment compared to DHC^WT^. The difference is abolished by nocodazole treatment and recovers after drug washout. ^∗∗∗^p < 0.001, ^∗∗^p < 0.01 in a Kruskal-Wallis test followed by paired Wilcoxon tests. n > 86 per condition, three independent experiments. (D) Still images from movies of DHC^WT^ and DHC^MT-B^ cells synchronized in G2 and treated with nocodazole (3.3 μM) for 30 min before washout. Time (t) is relative to start of the washout. Scale bar, 10 μm. (E) Quantification of the GFP intensity at microtubule-dependent foci in cells from (D) show that accumulation is greater and faster in DHC^MT-B^. n = 10 cells per condition, Mean ± SD shown. (F) Immunofluorescence of HeLa, DHC^WT^, DHC^MT-A^, and DHC^MT-B^ metaphase cells with antibodies against α-tubulin, GFP, and p150^Glued^. Scale bar, 5 μm. (G and H) The ratio of GFP (Dynein, G) or p150 (Dynactin, H) intensity at individual spindle poles divided by the corresponding intensity in the cytoplasm for DHC^WT^, DHC^MT-A^, and DHC^MT-B^ cells. Dynein and dynactin are enriched at spindle poles of DHC^MT-A/B^ cells. ^∗∗∗^p < 0.001, ^∗^p < 0.025 in a Kruskal-Wallis test followed by paired Wilcoxon tests. n > 120 spindle poles per condition from three independent experiments. (I) Cells imaged with DAPI (DNA) and α-tubulin antibodies (top row) or GFP (bottom row; displayed with a “fire” lookup table) showing representative prometaphase phenotypes in DHC^WT^, DHC^MT-A^, and DHC^MT-B^ cells. Scale bar, 5 μm. (J) Quantification of the average percentage of prometaphase cells with monopolar spindles in HeLa, DHC^WT^, and DHC^MT-A/B^ cells shows that mutation of the phi-dynein interface leads to an increase in mitotic defects. Mean ± SD is shown, ^∗∗∗^p < 0.001, ^∗∗^p < 0.01 in a paired Student’s t test. n > 235 cells from three experiments.

The data above show that activation of dynein movement in vitro involves a transition from phi to open-dynein followed by cargo adaptor/dynactin binding. We next sought to understand whether phi-dynein plays a role in dynein’s regulation in the cell. We made bacterial artificial chromosome (BAC) transgenes to express either N-terminally GFP-tagged wild-type dynein HC (GFP-wtDyn) or double-mutant dynein HC carrying the phi-disrupting mutations R1567E and K1610E (GFP-mtDyn). We generated clonal HeLa cell lines stably expressing GFP-wtDyn (DHC^WT^) or GFP-mtDyn (DHC^MT-A^ and DHC^MT-B^). Western blots with anti-GFP antibody (Figure 6A, top) show that the transgenes in DHC^WT^ and DHC^MT-B^ are expressed at similar levels. The dynein transgenes are expressed at similar or lower levels to endogenous dynein HC in all clonal cell lines (Figure 6A, bottom).

We initially characterized the DHC^WT^ and DHC^MT-B^ cell lines because of their similar expression levels. Both immunofluorescence staining (Figures 6B and 6C) and live-cell imaging (Figures 6D and 6E) of interphase cells showed that GFP-mtDyn accumulates more at the centrosomes than GFP-wtDyn. The difference in accumulation is abolished upon depolymerization of microtubules with nocodazole and returns following washout (Figures 6B–6E). These data suggest the phi-particle is involved in regulating microtubule-dependent localization of dynein to centrosomes in cells.

We also found that GFP-mtDyn accumulates more than GFP-wtDyn at the spindle poles of mitotic cells (Figures 6F and 6G). This effect is seen in both DHC^MT-B^ and DHC^MT-A^, suggesting the difference is not dependent on GFP-mtDyn expression level or isolated to one cell line. Immunostaining showed a concomitant enrichment of dynactin with GFP-mtDyn, suggesting that they accumulate as part of a dynein/dynactin complex (Figures 6F and 6H). This altered localization of GFP-mtDyn was accompanied by defects in mitotic spindle assembly (Figures 6I and 6J). Spindle morphology in DHC^WT^ cells was indistinguishable from control HeLa cells (Figure 6J). In contrast, both DHC^MT-A^ and DHC^MT-B^ cells displayed over 5-fold more mono-polar spindles, in which centrosomes are not separated (Figures 6I and 6J). This suggests that the phi-particle plays an important role in regulating dynein during mitosis.

## Discussion

**Figure 7:**
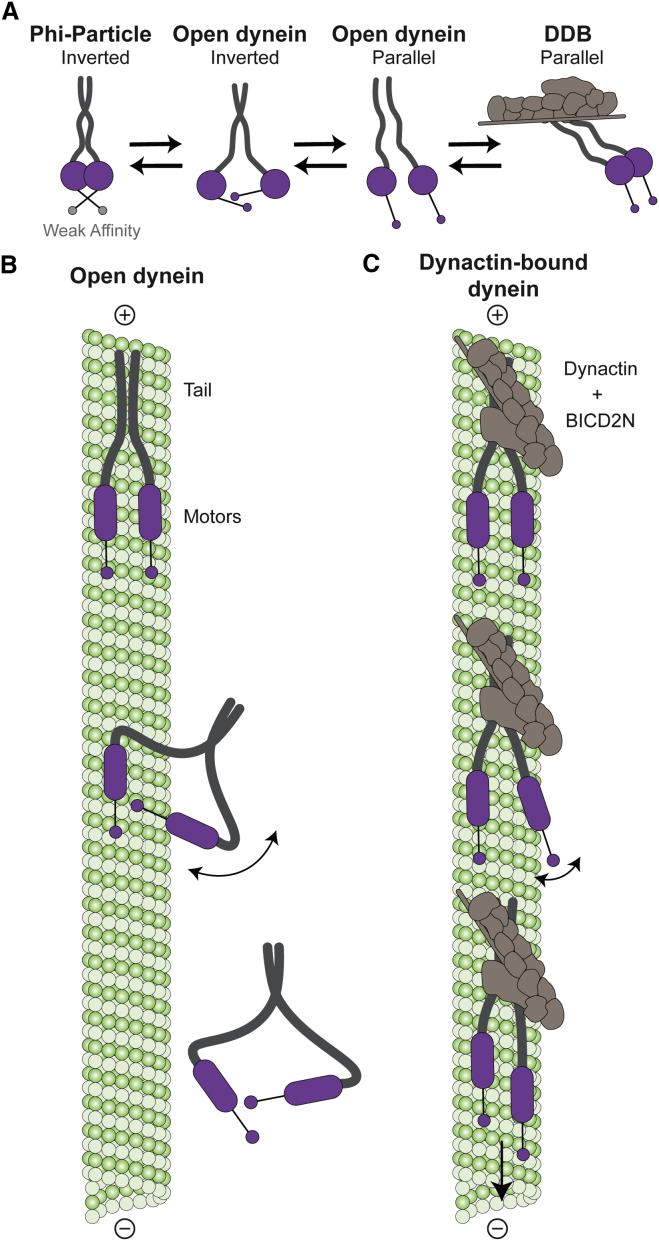
Model for How Dynactin Activates Processive Movement of Single Dyneins (A) Isolated dynein exists in either the phi-particle (motors dimerized, low affinity for the microtubule) or the open form (increased microtubule affinity). In open-dynein, there is an equilibrium between forms where the motor domain stalks are inverted or parallel. Dynactin and cargo adaptor stabilize open-dynein in its parallel state. (B) Open-dynein binds microtubules (green) with both motor domains (purple). During stepping the free motor domain is slow to rebind due to its preferred inverted orientation and large range of movement. Dissociation of dynein from the microtubule is likely. (C) In DDB, the dynein motor domains prefer a parallel orientation and are relatively constrained. DDB therefore moves processively because rebinding of a stepping motor is more likely than dissociation of the whole complex.

In the presence of cargo adaptors, dynein and dynactin assemble to form a transport machine that moves long distances along microtubules (McKenney et al., 2014, Schlager et al., 2014). Our work explains why dynein moves poorly in the absence of dynactin. We show that isolated dynein exists in two forms called phi- and open-dynein and that both are auto-inhibited (Figure 4H). We propose that the transition between them is an important part of dynein regulation (Figure 7A).

In phi-dynein, the motor domains are held together by interactions between their linker, AAA4, AAA5, and stalk domains (Figure 3A) and a contact between HCs in the tail (Figure 2E). The motor domains are locked with low affinity for microtubules (Figures 3B and 4C). Phi-dynein is also inhibited from binding dynactin (Figure 4E) because motor self-dimerization prevents it from undergoing the large conformational changes required (Movie S7; Figure 5E).

In contrast to phi-dynein, the motor domains in open-dynein are separate and quite flexible. However, we discovered open-dyneins can be in two major conformations (Figure 7A): an inverted form in which motor domains point toward each other and a parallel form in which they point in the same direction (Figure 5B). We suggest that open-dynein is weakly processive because the inverted form predominates (Figure 5B) and cannot move on microtubules. In our model (Figure 7B), the preference for the inverted form means that when open-dynein tries to take a step, the free motor domain is unlikely to be in the correct orientation for microtubule binding. It therefore cannot re-bind before release of the other motor domain from the microtubule. The tail is likely to determine open-dynein’s preference for the inverted form. This explains why artificially dimerized motor domains, which lack the tail, can be activated by forced separation (Torisawa et al., 2014).

Our work also shows how dynactin and BICD2N can activate dynein by driving the dynein motors into a parallel form (Figure 7A). It was previously observed that the motor domains are parallel on microtubules (Chowdhury et al., 2015). Here, we demonstrate that dynactin binding intrinsically pre-aligns dynein into a microtubule-binding ready conformation (Figure 5B). Dynactin also constrains the range of movement of the individual motor domains (Figure 5C). In our model (Figure 7C), the reduced range of separation and parallel orientation minimizes the search requirement of a stepping motor domain, making it more likely to re-bind before release of DDB from microtubules. The comparison of our DDB and the phi-particle cryo-EM structures shows that dynactin binding induces a rigid body twist of the HCs (Figure 5E; Movie S7). The change in motor orientation is thus driven directly by conformational changes in the dynein tail. Mutations in the dynein tail are associated with neurological diseases in both mice and humans (Schiavo et al., 2013) and are proposed to act via long-range, allosteric effects (Ori-McKenney et al., 2010). We speculate this is due to mutations interfering with the ability of dynactin to induce large rigid body rearrangements in the dynein tail.

The mechanism of dynein activation by dynactin described above may be conserved. In the case of S*accharomyces cerevisiae* dynein-1, isolated motors can move long distances on their own (Reck-Peterson et al., 2006), suggesting they are not auto-inhibited in the same way as mammalian dynein. However, dynactin increases the processivity of *S. cerevisiae* dynein by 2.5-fold (Kardon et al., 2009) and so may still promote motor domain re-orientation for full activation. In contrast, cytoplasmic dynein-2, which is involved in intra-flagellar transport, does not require dynactin for long-distance movement and so its activation is different from dynein-1. However, very recent work shows the dynein-2 motor domains self-dimerize in a phi-particle-like conformation, suggesting a common mechanism of auto-inhibition (Toropova et al., 2017).

Whereas this study focuses on how dynein HCs bind to the dynactin filament and BICD2N, other dynein subunits can also interact with dynactin and cargo adaptors. It will be interesting to understand the inter-play between these other contacts and the activation process described above. The IC N terminus binds to the CC1B coiled coil in dynactin’s p150^Glued^ subunit (Siglin et al., 2013, Tripathy et al., 2014), and antibodies against the IC N terminus inhibit dynein function in cells (Burkhardt et al., 1997). In vitro, a CC1B fragment stimulates the processivity of calf brain dynein attached to beads (Tripathy et al., 2014). An intriguing possibility is that the p150^Glued^/IC interaction provides a first step toward dynein activation before dynactin binds. In another example, the LIC C terminus binds the globular domain of the cargo adaptor Hook3 (Olenick et al., 2016, Schroeder and Vale, 2016). This interaction is required to form a processive dynein/dynactin/Hook3 complex. The LIC C terminus is disordered in our structure, but, given that the LICs are bound close to the phi-specific HC contact site, binding of the Hook3 dimer to both LIC C termini might break the tail contact site. The interaction of LIC and Hook3 may therefore facilitate the formation of open-dynein and thus an active dynein/dynactin complex.

Our current model is that dynein activation involves the transition from phi- to open-dynein. A key question is how important this transition is for proper dynein regulation. Our mutagenesis data show that disrupting the phi-particle increases dynein’s affinity for microtubules (Figure 4C) and dynactin (Figure 4E) as well as the number of processive DDB complexes (Figure 4G). This provides evidence that the phi-particle exists in solution and contributes to the pathway of dynein activation in vitro. Mutagenesis also suggests that the phi-particle plays a role in dynein regulation in cells. Stable, clonal cell lines expressing GFP-dynein HC with mutations at the phi interface show increased localization of dynein at centrosomes and spindle poles, where microtubule minus-ends are concentrated (Figure 6C and 6G). Mitotic spindle formation was often compromised in these cells, resulting in monopolar spindles in which spindle poles had not separated (Figure 6J). As dynein is required for inward forces on centrosomes that counteract spindle pole separation (van Heesbeen et al., 2014), this phenotype is consistent with phi-particle disruption causing mis-regulation of dynein activity. Taken together, our data support a physiological role for the phi-particle as part of the dynein activation pathway.

Intriguingly, we have also found that five mutations linked to human neuropathies lie at the phi interface. These mutations were identified in patients with malformation of cortical development (MCD) and/or spinal muscular atrophy with lower extremity predominance (SMA-LED) (Hertecant et al., 2016, Strickland et al., 2015). Mutations E1518K, R1567Q, and R1603T are in residues directly involved in electrostatic interactions at the phi interface (Figure S4E). Mutations G3658E and E3048K are directly adjacent to interacting residues (Figure S4E). Although other effects are possible (Hoang et al., 2017), the clustering of these mutations at the phi interface suggests they act by destabilizing the phi-particle and disrupting normal dynein regulation.

Dynein is the only cytoskeletal motor to require a large co-factor for its activity. Dynactin was discovered due to its ability to increase dynein movement (Schroer and Sheetz, 1991). Our structures explain why human dynein moves poorly on its own and demonstrates that dynactin can directly and allosterically activate its long-range movement.

## STAR★Methods

### Key Resources Table

**Table:** 

REAGENT or RESOURCE	SOURCE	IDENTIFIER
**Antibodies**		

Purified Mouse Anti-p150 [Glued]	BD Transduction Laboratories	Cat# 610473
Rabbit polyclonal anti-GFP	Abcam	Cat# AB6556; RRID: AB_305564
Purified Mouse anti-α-tubulin DM1α	Sigma-Aldrich	Cat# T6199; RRID: AB_477583
Rat dynein HC Antibody (R-325)	Santa Cruz Biotechnology	Cat# sc-9115; RRID: AB_2093483
Goat polyclonal anti-GFP	MPI-CBG Dresden	N/A
Rabbit polyclonal anti-pericentrin (PCNT)	Abcam	Cat# ab4448; RRID: AB_304461
Rat monoclonal anti-α-tubulin (YL1/2)	Santa Cruz Biotechnology	Cat# sc-53029; RRID: AB_793541
Sheep anti-mouse HRP	Amersham	Cat# NXA931-1ML
Donkey anti-rabbit HRP	Amersham	Cat# NXA934-1ML
Donkey anti-mouse DyLight 594	Bethyl	Cat# A90-337D4; RRID: AB_10630877
Donkey anti-rabbit DyLight 650	Bethyl	Cat# A120-208D5; RRID: AB_10630867
Donkey anti-goat Alexa Fluor 488	Jackson Imm. Res.	Cat# 705-545-147; RRID: AB_2336933
Donkey anti-rabbit Alexa Fluor 488	Invitrogen	Cat# A21206; RRID: AB_141708
Donkey anti-rat DyLight 594	Bethyl	Cat# A110-337D4; RRID: AB_10681669
Donkey anti-mouse Alexa Fluor 647	Invitrogen	Cat# A31571; RRID: AB_162542

**Bacterial and Virus Strains**		

*Escherichia coli* (One Shot Chemically Competent TOP10)	Thermofisher Scientific	Cat#C404010
*Escherichia coli* (DH10EMBacY)	Geneva Biotech	DH10EMBacY
*Escherichia coli* (HS996 clone CTD-2538J6)	MPI-CBG Dresden	Cat#MCB21917

**Chemicals, Peptides, and Recombinant Proteins**		

Biotin tubulin	Cytoskeleton	Cat#T333P-A
647-Tubulin	Cytoskeleton	Cat#TL670M-A
Unlabeled Tubulin	Cytoskeleton	Cat#T238P
SNAP-Cell TMR-Star	NEB	Cat#S9105S
Streptavidin	NEB	Cat#N7021S
Biotinylated poly(L-lysine)-[g]-poly(ethylene-glycol) (PLL-PEG-Biotin)	SuSoS AG	PLL(20)-g[3.5]- PEG(2)/PEG(3.4)- biotin(50%)
Insect-XPRESS Protein-free Insect Cell Medium with L-Glutamine	Lonza	Cat#12-730Q
Glucose Oxidase from *Aspergillus niger*	Sigma-Aldrich	Cat#G2133
Catalase from Bovine liver	Merck Millipore	Cat#219001
α-casein	Sigma-Aldrich	Cat#C6780
Glass cover slides (22 × 22 mm, Thickness No. 1)	VWR	Cat#631-0124
Double sided 15mm tape	tesa	Cat#05338
Pluronic F-157	Sigma-Aldrich	Cat#P2443
Glutaraldehyde solution	Sigma-Aldrich Aldrich	Cat#G5882
Fugene HD Transfection reagent	Promega	Cat#E2311
Complete, EDTA-free Protease Inhibitor Cocktail	Sigma-Aldrich	Cat#11873580001
Adenosine 5′-triphosphate disodium salt hydrate	Sigma-Aldrich	Cat#A2383
Nocodazole	Sigma-Aldrich	Cat#M1404
RO-3306	EMD-Millipore	Cat# 217699
L-glutamine	PAN Biotech	Cat#P04-80100
Penicillin / Streptomycin	PAN Biotech	Cat#P06-07100
Fetal Bovine Serum (FBS)	GIBCO	Cat#10270-106
G418 solution	SERVA	Cat#47995
Dulbecco’s modified Eagle medium (DMEM)	PAN Biotech	Cat#P04-03600
Trypsin/EDTA Solution	PAN Biotech	Cat#P10-023100
CO2 independent media	GIBCO	Cat#18045-054
Protease-inhibitor mix HP Plus	Serva	Cat#39107
ProLong Gold antifade reagent with DAPI	Life Technologies	Cat#P36935
ECL Prime Western Blotting Detection Reagent	GE Healthcare	Cat#RPN2232
Methanol	AppliChem Panreac	Cat#131091.1212
Benzonase Nuclease	Novagen	Cat#70746-10KUN

**Critical Commercial Assays**		

TSKgel G4000SWxL	TOSOH Bioscience	Cat#08542
TSKgel G4000SWxL guard column	TOSOH Bioscience	Cat#08543
IgG Sepharose 6 Fast Flow	GE healthcare	Cat#17-0969-02
Phusion High-fidelity DNA Polymerase	New England Biolabs	Cat#M0530S
Cre Recombinase	New England Biolabs	Cat#M0298L
Quickload Tap 2x master mix	New England Biolabs	Cat#M0271L
QIAprep Spin Miniprep Kit	Roche	Cat#27104
Effectene Transfection Reagent	QIAGEN	Cat#301425
NucleoBond Xtra BAC	Macherey Nagel	Cat#740436.25

**Deposited Data**		

Motor domains from human cytoplasmic dynein-1 in the phi-particle conformation (3.8Å).	This study	EMDB: EMD-3698; PDB: 5NUG.
Human cytoplasmic dynein-1 tail in the twisted state (8.4Å).	This study	EMDB: EMD-3703; PDB: 5NVS.
Human cytoplasmic dynein-1 tail in the parallel state (12Å).	This study	EMDB: EMD-3704.
Full length human cytoplasmic dynein-1 in the phi-particle conformation (15Å).	This study	EMDB: EMD-3705; PDB: 5NVU.
Human cytoplasmic dynein-1 bound to dynactin and an N-terminal construct of BICD2 (8.7Å).	This study	EMDB: EMD-3706; PDB: 5NW4.
Locally refined human cytoplasmic dynein-1 tail bound to dynactin and an N-terminal construct of BICD2 (12.4 Å).	This study	EMDB: EMD-3707; PDB: 5NW4.

**Experimental Models: Cell Lines**		

HeLa Kyoto	Hyman A. A., MPI-CBG Dresden	N/A

**Oligonucleotides**		

Forward GFP-tagging GAGTCGCGGCCGCCTTCTCATCGCTCCTGGAAGGTCCCGAGCGCGACACCATGGTGTCCAAGGGCGAGG	This study. Synthesized and HPLC purified by Sigma-Aldrich.	N/A
Reverse GFP-tagging ACTTCCAATCCGGCCGAGCCGTCCTCGCCGCCGCCGCCCCCGGGCTCCGATCCCTGAAAGTACAGGTTC	This study. Synthesized and HPLC purified by Sigma-Aldrich.	N/A
Forward counterselection cassette for R1567E TCTTCACAGGCAGTGCAGATATCAAGCACCTGCTGCCAGTGGAAACCCAGggcctggtgatgatggcggg	This study. Synthesized and HPLC purified by Sigma-Aldrich.	N/A
Reverse counterselection cassette for R1567E ACTAGCCGCTGGCAAATTTGGCTCTCTGGCTGGAGGCCATACCTCTGAAATTAGCCCTCCCACACATAAC	This study. Synthesized and HPLC purified by Sigma-Aldrich.	N/A
Rescue oligonucleotide for R1567E ACTAGCCGCTGGCAAATTTGGCTCTCTGGCTGGAGGCCATACCTCTGAAACTCCTGGGTTTCCACTGGCAGCAGGTGCTTGATATCTGCACTGCCTGTGAAGA	This study. Synthesized with phosphothioate bonds at the 2 first 5′ positions and PAGE purified by Sigma-Aldrich.	N/A
Forward counterselection cassette for K1610E ACATCCAGGGAGTACAGAGGTCTCTGGAAAGATTGGCAGACCTGCTAGGAggcctggtgatgatggcggg	This study. Synthesized and HPLC purified by Sigma-Aldrich.	N/A
Reverse counterselection cassette for K1610E GGGAAAGATGACCGCTCTCTTTCCAGATATTCTCCCAATGCTTTCTGGATTTAGCCCTCCCACACATAAC	This study. Synthesized and HPLC purified by Sigma-Aldrich.	N/A
Rescue oligonucleotide for K1610E GGGAAAGATGACCGCTCTCTTTCCAGATATTCTCCCAATGCTTTCTGGATTTCTCCTAGCAGGTCTGCCAATCTTTCCAGAGACCTCTGTACTCCCTGGATGT	This study. Synthesized with phosphothioate bonds at the 2 first 5′ positions and PAGE purified by Sigma-Aldrich.	N/A

**Recombinant DNA**		

pDyn1 (SNAPf-His-ZZ-LTLT- DYNC1H1 in pACEBac1)	(Schlager et al., 2014)	N/A
pDyn2 (DYNC1I2, DYNC1LI2, DYNLt1, DYNLL1, DYNLRB1 in pIDC)	(Schlager et al., 2014)	N/A
Bacterial Artificial Chromosome CTD-2538J6	MPI-CBG Dresden	Cat#MCB21917
pABRG	(Bird et al., 2011)	N/A
Rpsl-Amp counterselection cassette	(Bird et al., 2011)	N/A
TH0478-R6Kamp-hNFLAP	(Poser et al., 2008)	N/A
CTD-2538J6 N-FLAP-DYNC1H1	This study	N/A
CTD-2538J6 N-FLAP-DYNC1H1 R1567E and K1610E	This study	N/A

**Software and Algorithms**		

Gautomatch	Kai Zhang	http://www.mrc-lmb.cam.ac.uk/kzhang/Gautomatch/
Gctf	(Zhang, 2016)	http://www.mrc-lmb.cam.ac.uk/kzhang/Gctf/
EM scripts	Kai Zhang	http://www.mrc-lmb.cam.ac.uk/kzhang/useful_tools/scripts/
MotionCorr	(Li et al., 2013)	http://cryoem.ucsf.edu/software/driftcorr.html
Eman2	(Tang et al., 2007)	http://blake.bcm.edu/emanwiki/EMAN2
RELION-1.4	(Scheres, 2012)	http://www2.mrc-lmb.cam.ac.uk/relion/index.php/Main_Page
Coot	(Emsley et al., 2010)	http://www2.mrc-lmb.cam.ac.uk/personal/pemsley/coot/
Chimera	(Pettersen et al., 2004)	https://www.cgl.ucsf.edu/chimera/
REFMAC	(Brown et al., 2015)	https://www2.mrc-lmb.cam.ac.uk/groups/murshudov/content/refmac/refmac.html
Pymol	PyMOL	http://www.pymol.org
Molprobity	(Chen et al., 2010)	http://molprobity.biochem.duke.edu/
Phyre2	(Kelley et al., 2015)	
FIJI	(Schindelin et al., 2012)	https://fiji.sc/
GraphPad Prism	GraphPad	http://www.graphpad.com/scientific-software/prism/
R version 3.2.3	R-project	https://www.R-project.org/Licenses/

**Other**		

UltrAuFoil R 1.2/1.3, 300 mesh, Gold	Quantifoil	N/A
Carbon film 400 mesh Copper grid	Agar Scientific	CF400-Cu
Amicon Ultra-15 Centrifugal Filter Unit	Merck-Millipore	Cat#UFC910024
CellASIC ONIX Complete Perfusion System	Merck-Millipore	Cat#EV262
CellASIC ONIX switching plate mammalian cells (4 chamber)	Merck-Millipore	Cat#M04S-03-5PK
Amersham Hyperfilm ECL	Amersham	Cat#28906835

### Contact for Reagent and Resource Sharing

Further information and requests for reagents should be directed to and will be fulfilled by the Lead Contact, Andrew P. Carter (cartera@mrc-lmb.cam.ac.uk).

### Experimental Model and Subject Details

Sf9 (*Spodoptera frugiperda*) cells were maintained in suspension culture in Insect-XPRESS media (Lonza) at 124rpm, 27°C.

HeLa Kyoto and derivative cell lines were maintained at 37°C and 5% CO_2_ in Dulbecco’s modified Eagle medium (DMEM) supplemented with 10% FBS (GIBCO), 2mM L-Glutamine (PAN Biotech), Penicillin 100 U/ml and Streptomycin 0.1 mg/ml (PAN Biotech). For transgenic lines harboring G418 resistance, 300 μg/ml G418 was added to the culture medium.

### Method Details

#### Cloning and plasmid production

Mutations were made in the pDyn1 plasmid (Schlager et al., 2014) which contains the codon optimized sequence of DYNC1H1 with an N-terminal SNAPf-His-ZZ-2xTevCleavage tag in the pACEBac1 vector. Two point mutations (E1518K and R1567K) were made by primer-based site-directed mutagenesis. Forward and reverse primers containing the mutation and flanking 15 base pairs were designed for each site and two PCR reactions were set up. The first amplified the region between the two mutation sites to form an insert and the second amplified the rest of the plasmid backbone. Phusion polymerase (New England Biolabs) was used according to manufacturer’s guidelines. Gibson assembly was used to seamlessly fuse the insert and backbone, which each contain the mutated nucleotides in their overlapping, 15bp overhangs. Whole plasmid sequencing, performed by the CCIB DNA Core Facility at Massachusetts General Hospital (Cambridge, MA), confirmed that only the desired mutations were present. The mutated pDyn1 was subsequently fused to pDyn2 (containing all accessory chains) (Schlager et al., 2014) using an in vitro cre reaction (New England Biolabs) to form the mutated pDyn3. The presence of all 6 dynein genes was verified by PCR, using Quickload Taq 2x master mix (New England Biolabs) according to manufacturer’s guidelines.

#### Insect cell expression of dynein and BICD2N

Full-length dynein (wild-type or mutant) was expressed in Sf9 cells using the multiBAC system as previously described (Schlager et al., 2014). pDyn3 was transformed into EmBacY cells (Multibac) by heat shock, and successful integrants were identified in a blue/white selection screen. Single, white colonies were inoculated into 2xTY media supplemented with 7 μg/ml gentamycin, 10 μg/ml tetracycline and 50 μg/ml kanamycin and grown overnight at 37°C. For bacmid preparation, bacteria were pelleted at 4000 rcf for 5 min and resuspended in 0.3 mL QIAGEN miniprep buffer P1, followed by 0.3mL P2 buffer. After 5 min incubation, 0.4 mL P3 buffer was added and incubated on ice for 6 min. After 10 min centrifugation at 20000 rcf, the supernatant was added to 0.8 mL ice cold isopropanol and incubated for 1hr on ice. Bacmid DNA was pelleted for 10min at 13000 rcf. The pellet was washed three times in 0.75 mL 70% ethanol before being air-dried for 1 min and resuspended in EB buffer to a concentration of 1 μg/μl.

Fresh bacmid DNA was transfected into 2 mL Sf9 cells at 0.5x10^6^ cells/ml using FuGene HD (Promega) according to manufacturer’s protocol. Three days later, 1ml of the transfected culture was used for P2 infection of a 50 mL culture of Sf9 cells (at 1x10^6^ cells/ml). After three days, the P2 virus was harvested by centrifugation of the cells at 4000 rcf for 15 min and collection of the supernatant. P2 virus was stored at 4°C in the dark until required. Protein expression was induced by addition of 5 mL of P2 virus per 500 mL of Sf9 cells (at 1-2x10^6^ cells/ml). 2-3L of cells were infected at a time. After three days, cells were harvested by centrifugation at 4000 rcf for 10 min at 4°C. The pellet was resuspended in ice-cold PBS and pelleted again. The supernatant was discarded and pellet flash frozen in liquid nitrogen before being stored at −80°C.

Residues 1-400 of mouse BICD2 with GFP (GFP-BICD2N) or SNAPf (SNAPf-BICD2N) N-terminal tags were also expressed in Sf9 cells. Their plasmid production is described in (Schlager et al., 2014).

#### Purification of full length dynein or BICD2N

For recombinant protein purification, frozen pellets from 500 mL insect cell culture were thawed on ice in 50 mL lysis buffer (50 mM HEPES pH 7.4, 100 mM NaCl, 10% (v/v) glycerol, 1 mM DTT, 0.1 mM ATP, 2 mM PMSF) with 1 protease inhibitor tablet added per 50ml (Complete-EDTA Free Protease Inhibitor tablet, Roche Applied Science). Cells were lysed in a 40 mL dounce-type tissue grinder (Wheaton) using 15-25 strokes. The lysate was cleared by ultracentrifugation (503000 rcf, 45 min, 4°C, Type 70 Ti Rotor, Beckman Coulter) and incubated with 1.5-3 mL IgG Sepharose 6 Fast Flow beads (GE Healthcare), pre-equilibrated in lysis buffer, for 1-4 hr on a roller. After incubation, the beads were applied to a gravity flow column and washed with 150 mL lysis buffer. If fluorescent labeling of the SNAPf tag was required, SNAP-Cell TMR-Star (New England Biolabs) was suspended to 1 mM in 100% DMSO (Sigma-Aldrich), added to the dynein coated beads to a final concentration of 10 μM and incubated on a roller for 1-2 hr before continuing the purification. Dynein coated beads were washed in 200 mL TEV buffer (50 mM Tris–HCl pH 7.4, 150 mM KAc, 2 mM MgAc, 1 mM EGTA, 10% (v/v) glycerol, 0.1 mM ATP, 1 mM DTT). The beads were resuspended in 2-5 mL TEV buffer containing 100 μL TEV protease (4mg/ml) and incubated on a roller overnight at 4°C. The cleaved protein was separated from the beads by collecting the flow-through of a gravity-flow column and concentrated to a volume of 150-250 μL using an Amicon Ultracel concentrator (Merck- Millipore) with a 100K molecular weight cut-off. TEV protease was removed by size-exclusion chromatography (SEC) using a TSKgel G4000SWXL column with a TSKgel SWXL guard column (TOSOH Bioscience) equilibrated in GF150 buffer (25 mM HEPES pH7.4, 150 mM KCl, 1mM MgCl2, 5 mM DTT, 0.1 mM ATP). Peak fractions were collected, pooled and concentrated to a concentration of 0.5 – 3 mg/ml. If frozen, solutions were supplemented with glycerol to a final concentration of 10% (v/v) before snap freezing in liquid nitrogen and storage at −80°C. All purification and concentration steps were performed at 4°C.

BICD2N purification was performed as described above but with the following modifications. 1 mL of pre-equilibrated IgG Sepharose 6 FastFlow beads (GE Healthcare) were added to the cleared lysate of a 250 mL insect cell culture pellet. After washing and TEV cleavage, the protein solution was concentrated to 3 – 6 mg/ml and 50 μL aliquots were snap frozen in in liquid nitrogen and stored at −80°C. TEV protease was removed from thawed aliquots by size size-exclusion chromatography as described above. Peak fractions were pooled and concentrated to 0.5 – 6 mg/ml using an Amicon Ultracel concentrator (Merck- Millipore) with a 100K molecular weight cut-off and frozen as above.

#### Purification of dynactin

Large scale purification of endogenous dynactin from pig brains was carried out essentially as previously described (Urnavicius et al., 2015). Fresh pig brains were obtained from the local butcher, Leech & Sons (Melbourne, UK) and placed in ice cold phosphate buffered saline (PBS) shortly after slaughter. Brains were washed twice in PBS and the brain stem and large blood vessels were removed. Each brain was then washed in homogenization buffer (HB) (35 mM PIPES-KOH pH 7.2, 1 mM MgSO4, 0.2 mM EGTA, 0.1 mM EDTA, 1 mM DTT), flattened and snap frozen in liquid nitrogen before being stored at −80°C. For each purification, three brains were broken up and added to 400 mL HB supplemented with 3 protease inhibitor tablets (Complete-EDTA Free, Roche Applied Science) and 1.6 mM PMSF (diluted from 200mM stock in dry ethanol). The brains were homogenized at room temperature in a Waring blender, using four 5 s pulses initially then 15 s pulses interspersed by 15 s waits. The temperature of the lysate remained below 4°C in this and subsequent steps. Once thawed completely, the lysate was cleared in a TLA16.250 rotor (Beckman Coulter) at 38000 rcf for 15 min and again in a Ti45 rotor (Beckman Coulter) at 235000 rcf for 50 min. The resulting supernatant was the filtered through Glass fiber (Sartorius) and 0.45 μm syringe filters (Elkay) before being loaded onto 300 mL of SP-Sepharose Fast Flow (GE Healthcare), packed in an XK 50/30 column (GE Healthcare) and equilibrated in buffer A (HB buffer with 0.1 mM Mg-ATP). Unbound sample was washed from the column with 4 column volumes (CV) of buffer A. The remaining proteins were fractionated in a two phase salt gradient: 0% to 25% buffer B (HB buffer with 0.1 mM Mg-ATP and 0.5 M KCl) in 3 CV and 25% to 100% buffer B in 1 CV. Fractions containing dynactin typically elute at 12% buffer B (20 mS/cm) and were initially identified by western blot with p150^Glued^ antibody (BD Transduction Laboratories). This ∼270 mL of eluate was loaded onto a MonoQ HR 16/10 column (GE Healthcare) equilibrated in 95% HB buffer and 5% buffer C (HB buffer and 1 M KCl) and then the column was washed with 10 CV 5% buffer C. A three phase linear gradient was used to elute dynactin from the column: 5% to 15% buffer C in 1 CV, 15% to 35% buffer C in 10 CV and then 35% to 100% buffer C in 1 CV. Dynactin containing fractions typically elute at 29% buffer C and were pooled and concentrated in an Amicon Ultracel concentrator (Merck-Millipore) with a 100K molecular weight cut-off to 1-2ml. Size-exclusion chromatography was then performed as described above. The resulting, dynactin containing fractions were pooled and concentrated to the desired concentration (0.5-1.5mg/ml for single molecule studies, 3-4mg/ml for dynein-dynactin-BICD2N complex assembly) before snap freezing in liquid nitrogen.

#### Flow chamber and microtubule preparation

Glass coverslips (22 × 22 mm, Thickness No. 1) were cleaned by 30 min sonication in 3M NaOH, thorough washing in dH_2_O, and then 30 min sonication in Piranha solution (60% (v/v) sulphuric acid + 40% (v/v) hydrogen peroxide). Clean slides were again washed in dH_2_0 and were stored in dH_2_0 for up to a week. Shortly before use, slides were air-dried with N_2_ gas. Flow chambers were created by placing two strips of double-sided tape (tesa) parallel on a glass slide and placing a cleaned coverslip above, creating a channel around 15 μL in volume.

Biotinylated, Alexa 647-labeled microtubules (Cytoskeleton Inc.) were polymerized by mixing 1 μL Alexa-647 tubulin (2 mg/mL), 2 μL Biotinylated-tubulin (2 mg/mL) and 2 μL unlabeled tubulin (11 mg/mL) with 5 μL BRB80 (80mM PIPES pH6.9, 1mM MgCl2, 1mM EGTA), on ice. To this, 10 μL of 2X Polymerization buffer (20% DMSO, 2mM MgGTP in 1xBRB80) was added and incubated at 37°C for 45 min (final concentrations are 1 μM Alexa-647 tubulin, 2 μM Biotinylated tubulin, and 22 μM unlabeled tubulin). 100 μL BRB80 containing 10 μM Taxol (BRB80-T) (warmed to 37°C) was added to stabilize the microtubules, followed by centrifugation for 9 min at 16000 rcf on a benchtop centrifuge (room temperature). The microtubule pellet was resuspended in 100 μL BRB80-T (by gentle flicking to avoid MT severing) and spun again. The second pellet was resuspended in 50 μL BRB80-T and stored at room temperature, wrapped in foil, for up to a week.

#### TIRF microscopy of single dynein complexes

To assemble dynein-dynactin-BICD2N (DDB) complexes for single molecule assays, 1 μL of fully purified TMR labeled wild-type dynein (wtDyn) or phi-interface mutant dynein (mtDyn) at a concentration of 200nM was mixed with 2ul of dynactin and 1ul SNAPf-BICD2N to achieve a molar ratio of 1:2:2. After 15 - 30 min incubation on ice, the complex was diluted to 10 μL in TIRF assay buffer (TAB) (25mM HEPES pH7.2, 5mM MgSO4, 1mM EGTA, 1mM DTT, 10 μM Paclitaxel).

Flow chambers were prepared by passivation with 15 μL of 1% (w/v) Pluronic F-127 (Sigma-Aldrich). This solution was washed through with 15 μL TAB, followed by 15 μL 0.5mg/mL PLL-PEG-Biotin (SuSoS AG), 15 μL TAB, 15 μL 1mg/mL Streptavidin (New England Biolabs) and 15 μL TAB. The required dilution of microtubule stock in BRB80-T was determined empirically on each day. The microtubule dilution was made fresh for each slide and was typically around 10-fold. The diluted microtubules were flowed into the chamber, incubated for 1-5 min and washed with 15 μL TAB then 30 μL TAB supplemented with 1.25 mg/mL α-casein (Sigma-Aldrich). The following reagent dilutions were made in TAB: 20 mM MgATP (100mM stock), 2.86M 2-mercaptoethanol, 9% Glucose (w/v) (45% w/v stock), Gloxy mix (30 mg/mL Glucose oxidase (Sigma-Aldrich), 4 mg/mL Catalase (Merck Millipore)). The motility mixture was made fresh for each slide by adding 1 μL of each of these reagents, 1 – 2 μL diluted DDB complex or dynein alone and 15 μL TAB supplemented with 1.25 mg/mL α-casein and 25mM KCl (final concentrations). 15 μL of the motility mixture was flowed into the chamber and all imaging was completed within 5 min of its addition.

Imaging was performed at room temperature on a Nikon total internal fluorescence microscope, with a 100x objective lens (Nikon, 1.49 NA Oil, APO TIRF). Illumination was provided by a 150mW 561nm laser (Coherent Sapphire) and a 100mW 641nm laser (Coherent Cube). Movies were collected with 100ms frames and 25 ms delay (8fps) unless otherwise stated, using a back-illuminated EMCCD camera (iXonEM+ DU-897E, Andor, UK) controlled with uManager software (http://micro-manager.org/wiki/Micro-Manager).

#### Microtubule-gliding assays

Microtubules were polymerized as for single-molecule assays, except biotinylated-tubulin was omitted, and the final concentrations of Alexa-647 and unlabeled tubulin were 3uM and 11uM respectively. Unlabeled wtDyn and mtDyn was used fresh, diluted to a concentration of 200nM. 15uL of dynein was added to a flow chamber and incubated at 25°C for 2 min. 15uL TAB supplemented with 1.25mg/ml α-casein was then flowed through the chamber. The assay mixture was assembled by adding 1 μL polymerized microtubules, 1uL 100mM MgATP, 1uL 45% (w/v) glucose, 1uL Gloxy mix (150mg/ml Glucose oxidase, 20mg/mL Casein) to 100uL of TAB supplemented with 1.25mg/ml α-casein and 75mM KCl. 15uL of the assay mixture was flowed into the chamber. After 3 min the chamber was imaged. Imaging was performed with 20ms exposures taken at 3 s intervals, one pixel = 0.16 × 0.16μm.

#### DDB preparation for SEC and cryo-EM

To analyze the amount of DDB complex formation by size exclusion chromatography (SEC), 500 nM fully purified dynein, 1 μM pig dynactin and 4 μM SNAPf-BICD2N (dimer) were mixed in a volume of 80 μL and incubated on ice for 15 min before SEC was carried out as described above. The elution volume of each species was determined by running fractions corresponding to different peaks on SDS-PAGE (Novex 4%–12% Bis-Tris precast gels run in MOPS buffer - Life Technologies) stained with Instant Blue Stain (Expedeon) or SYPRO-Ruby (BioRad), according to manufacturer’s instructions. The elution volume individual components (dynein, dynactin and BICD2N) was confirmed by separate SEC runs. Running the same gel in MES buffer allowed visualization of individual dynein light chains.

For preparation of DDB samples for electron microscopy, 270 nM dynein, 540 nM dynactin and 5 μM GFP-BICD2N were diluted to 100 μL in GF150 buffer and incubated on ice for 15 min. 10 μL of 0.1% (v/v) glutaraldehyde (Sigma-Aldrich), diluted in GF150 was added and incubated for a further 15 min. The cross-linking reaction was quenched by addition of 10 μL 1M Tris-HCl pH 7.4 before SEC. The DDB containing fractions (identified as above but using SYPRO Ruby Gel Stain (Bio-Rad)) were pooled and concentrated to a volume of 30 μl.

#### Negative stain EM of phi-dynein

For negative stain grid preparation, dynein samples were diluted to a concentration of ∼0.05 mg/ml in GF150 buffer. 400 mesh copper grids coated with a continuous carbon support layer (Agar scientific) were treated by plasma cleaning for 25 s using a 9:1 mixture of Ar and O_2_in a Fischione NanoClean (Model 1070). 3ul of the sample was applied to the grid and incubated for 1 min before 2% Uranyl acetate stain was added. For 2D analysis and initial model building of the phi-particle, 3000 micrographs were manually collected on 120kV FEI Spirit T12 microscope equipped with Gatan 2K × 2K CCD (model 984), at a nominal magnification of 30,000X with the digital pixel size 3.3Å.

Grids were made and imaged in the same way for statistical analysis of motor domain orientation and analysis of the proportions of phi- and open-dynein in wtDyn and mtDyn preparations (details in Quantification and Statistical Analysis). When testing the effect of nucleotide addition on phi-particle formation, we prepared wtDyn as described above but omitted ATP from the GF150 buffer. The complex eluted from the SEC column in the same fraction as when run in GF150 with ATP. The sample was then pooled, concentrated and diluted to ∼0.05 mg/ml in GF150 with either no nucleotide or the desired nucleotide present (ATP, ATP.Vi, ADP and ADP.Vi were tested at 0.1mM and 0.5mM final concentration). Grids were made as above and quantification performed and described below.

For particle picking, single phi-particles were manually selected and low-pass filtered to 50Å, then used as a template to automatically select ∼500 particles from 10 micrographs using Gautomatch (developed by K.Z, http://www.mrc-lmb.cam.ac.uk/kzhang/Gautomatch/). These particles were classified into 10 distinct 2D class averages in RELION-1.4 (Scheres, 2012) and then used as templates to pick all particles from 3000 negative stain micrographs in Gautomatch. Performing several cycles of 2D classification ensured that each class contained only relatively similar particles. All subsequent processing was performed in RELION-1.4 (Scheres, 2012) unless otherwise stated.

For 3D classification of the phi-particle, a map of dynactin was low pass filtered to 100Å and used as an initial model. This worked well because dynactin and the phi-particle share a thin, elongated shape of similar size. A 30Å reconstruction of phi-particle was generated after several iterative classifications. This map was then used as an initial model for 3D refinement and classification of the cryo-EM dataset.

#### Cryoelectron microscopy

In order to obtain a sufficient proportion of phi-particles on cryo-EM grids, freshly purified dynein in GF150, at a concentration of ∼0.15mg/ml, was used directly after SEC. Quantifoil R1.2/1.3 Au grids were prepared by coating with homemade thin carbon layers. These were made in an Edward carbon evaporator (Auto 306) on mica to a thickness of between ∼50 nm. Carbon-coated grids were plasma cleaned for 25 s using a 9:1 mixture of Ar and O_2_ gas. 3 μL of the sample was incubated on the grid for 30 s and then blotted, using a Vitrobot IV (FEI), for 2-5 s at 100% humidity and 4°C. All grids were stored in liquid nitrogen dewars before loading to the electron microscope.

Screening of cryo-EM conditions was performed on a 120kV FEI Spirit T12 microscope. Initial datasets were collected on FEI 300kV Polara microscope with Falcon III detector. We obtained preliminary 2D class averages and confirmed the possibility of successful 3D reconstruction with these datasets. Due to the flexibility, heterogeneity and severe orientation preference of the phi-particle, large datasets were collected to obtain sufficient views with relative stable conformations. Despite the large size of full-length dynein, contrast was limited by its thin elongated shape and an additional carbon support which was required to prevent the complex from falling apart. We therefore only selected holes that allowed us to obtain both sufficient contrast and intact particles. Datasets were collected on FEI 300kV Titan Krios microscopes equipped with Falcon II or K2 summit detectors over 8 separate sessions. A summary of the imaging conditions of all the datasets are listed in Table S1.

#### Structure of the dynein phi particle

To begin processing the cryo-EM dataset, all electron micrograph movies were aligned by Motioncorr (Li et al., 2013). Only movies with relatively small and smooth movements for all frames were used for further analysis. Any movies with abnormal normalization or background, heavy contamination, thick ice, low contrast or large drift were discarded.

CTF parameters of the averaged movie frames from Motioncorr were automatically determined by Gctf (Zhang, 2016). The equiphase averaging (EPA) method in Gctf was used to estimate the maximum information limit for each micrograph. Only micrographs which contained CTF information higher than 4Å were used in subsequent processing. We also only used micrographs with a defocus range of 1.5-5.0 μm for Falcon II datasets or 1.0-3.5 μm for K2 datasets.

Automatic particle picking was performed using Gautomatch, on phase-flipped micrographs generated by Gctf. The templates for picking were generated in RELION from 2D class averages of negative stain EM images of the phi particle. Gautomatch was run using a low threshold (0.1 cross-correlation) to pick as many particles as possible. The output is a list of particle coordinates with the associated cross-correlation coefficient. Micrographs from each dataset were then empirically split into 3-5 groups according to the contrast observed. The correlation cut-off parameter was refined for each group of micrographs to optimize particle selection. Selection of good particles was performed on output coordinates from Gautomatch using the script box_filter.com (http://www.mrc-lmb.cam.ac.uk/kzhang/useful_tools/scripts/). We then used RELION to extract the particles from the original micrographs rather than the phase-flipped images.

These selected particles were subsequently split into sub-datasets (∼50,000 particles per sub-dataset, total particle numbers for each dataset are given in Table S1) for 2D analysis in RELION. Initial 2D classification of each sub-dataset into ∼20 classes could not sufficiently separate all of the distinct conformations and orientations of the phi-particle, due to its extreme flexibility. To improve the quality of the 2D averages, sub-classification was performed on each class that contained good averages. Sub-classification allowed us to further separate the smaller conformational changes within each major class. In addition to the initial 2D classification a subsequent 3-6 cycles of 2D sub-classification was required to remove most bad particles.

High quality particles were re-extracted from the original movies corrected by a composite weighting function. This process involved the following steps. Coordinates of good particles were passed to Gctf for accurate local CTF refinement (Zhang, 2016). A smooth analytic function was fitted, by least-squares, to the discrete drifts estimated by Motioncorr for each movie frame. This smooth function estimates the physical drift in the movie more realistically because beam-induced or stage drift is assumed to be locally continuous. A composite weighting function for the Fourier coefficients of each original movie frame was estimated using the combination of smooth drift, radiation damage (Grant and Grigorieff, 2015) and CTF quality. The scripts used to generate this composite weighting function are under development and available on request (kzhang@mrc-lmb.cam.ac.uk). The weighted and averaged particles were used for all subsequent processing. No further processing required the movie stacks.

3D classification and refinement of the full-length dynein led to a 15Å reconstruction. It was not possible to further improve the maps because of the continuous relative rotation between motor domain and tail. We therefore solved the structure of the motor domain and tail separately and used the 15Å map as a reference to assemble the two parts together. We re-centered and re-extracted particles so that they contained only the motor domain or tail region, using a box size of 400 for motor domains and 288 for the tail. We processed these datasets separately in the following 2D and 3D classification and refinement steps.

2D classification was performed to select high quality motor domains. Then, one cycle of automatic 3D refinement using C2 symmetry was performed to estimate the alignment parameters. These parameters were fixed during subsequent 3D classification to reduce the uncertainty and improve the classification. The particles in the major class were used for subsequent 3D refinement and this generated a map of the motor dimer at 3.8Å resolution. We also performed focused classification and refinement of a single motor domain to improve the density map of the stalk and MTBD. We used the motor monomer map to build a portion of the stalk that wasn’t clearly resolved in the motor dimer map.

The tail was processed using similar strategy but more cycles of 2D and 3D classification were performed to remove low quality particles. Although we collected extensive data, the intrinsic flexibility of the tail prevented its reconstruction at near-atomic resolution. 3D classification worked best when 8 classes were used. The major flexibility in these classes came from the tail N terminus, neck and Robl light chain but was not limited to these regions. The N terminus shows a dominant twisted conformation in the major class (44%) and a second major class with parallel conformation (18%). The best class with a twisted N terminus reached a final resolution of 8.4Å and the parallel class reached 12.8Å.

#### Structure determination of the DDB complex

DDB samples were prepared as described and diluted to a concentration of 0.15mg/ml before grids were made using the same conditions as for dynein alone.

Projections from a previous structure of the dynein tail bound to dynactin and BICD2N (TDB, EMD-2860) were used as templates to pick DDB particles using Gautomatch. 2D and 3D classification were performed after masking out the motor domains which are extremely flexible. We obtained an 8.7Å density map of the DDB which closely resembled the TDB structure but contains significant additional density for the HC and LIC.

In order to resolve the dynein tail within DDB further, we subtracted the density of dynactin from each particle (Bai et al., 2015) using the previously determined structure of dynactin (PDB: 5ADX) as a mask. Using the subtracted map for further 2D and 3D analysis significantly improved the density in the dynein tail region. We resolved the nearly complete tail in DDB to 12Å and can rigid body fit HC helix bundles (except bundle 9) into to the density map.

#### Model building and refinement

A de novo model of the 3.8Å motor domains was manually built using COOT (Emsley et al., 2010). The model was refined using REFMAC (Brown et al., 2015). Statistics of the model building and refinement are summarized in Table S1. Helical bundles 2-8 in the phi-dynein tail were manually built by fitting poly-alanine α helices into density using COOT. Helical bundle 1 and the N-terminal domain were obtained from a previous 5Å crystal structure (PDB: 5AFR) of the N terminus of the dynein tail (Urnavicius et al., 2015) and fit into density. A homology model of helical bundle 9 was built using the Phyre2 server (Kelley et al., 2015). Homology models for the human dynein intermediate chain WD40 domain and human dynein light intermediate chain were also obtained from Phyre2. These models were fit into tail of phi-particle as rigid bodies using UCSF chimera (Pettersen et al., 2004). A de novo model was manually built into the 3.8Å map of the phi-dynein motor domains using COOT (Emsley et al., 2010). The model was refined using REFMAC (Brown et al., 2015). Statistics of the model building and refinement are summarized in Table S1. A model for the dynein tail was assembled using the 8.4Å map of the phi-dynein tail. Helical bundles 2-8 in the dynein tail were manually built by fitting poly-alanine α helices into density using COOT. Helical bundle 1 and the N-terminal domain were obtained from a previous 5Å crystal structure (PDB: 5AFR) of the N terminus of the dynein tail (Urnavicius et al., 2015) and fit into density. A homology model of helical bundle 9 was built using the Phyre2 server (Kelley et al., 2015). Homology models for the human dynein intermediate chain WD40 domain (based on PDB: 1ERJ) and human dynein light intermediate chain (based on PDB: 4W7G) were also obtained from Phyre2. These models were fit into tail of phi-particle as rigid bodies using UCSF chimera (Pettersen et al., 2004).

Helix bundles 1-4 in DDB were directly built into the 8.7 Å density map while dimerization domains of the HC, ICs and Robl were unambiguously fit into the density as rigid bodies. Helix bundles 5-8 and LICs were fit into the 12 Å density map as rigid bodies. The position of helix bundle 9 was estimated according to the position of bundle 8 and also the characteristic curvature of the neck region. This was further conformed from locally refined 2D averages.

#### Map visualization and analysis

The electron density maps and models were visualized in Chimera (Pettersen et al., 2004) and Pymol (http://www.pymol.org). Density maps were segmented within Chimera using the fitted homology models (ICs, LCs and LICs) or manually built helices (HCs). The interaction interfaces were analyzed in COOT (Emsley et al., 2010). Figures and movies were made in Pymol and Chimera. Local resolution of the maps was analyzed by ResMap using confidence level (p value) of 0.05 and 0.5Å step size (Kucukelbir et al., 2014). For most of the regions in tail, we used the 8.4Å map. But the density of the N terminus of HCs and LCs are not clearly visible if the map is automatically sharpened to 8.4Å using RELION post-processing. Therefore, the unsharpened map was used to locate and visualize those regions.

#### Bacterial artificial chromosome recombineering

The *Escherichia coli* HS996 clone CTD-2538J6 harboring a bacterial artificial chromosome (BAC) encompassing the entire DYNC1H1 gene was obtained from the Hyman lab at the MPI-CBG Dresden. The pABRG plasmid (Bird et al., 2011) was electroporated into CTD-2538J6 and used for the expression of Redβ and γ proteins in all subsequent Red E/T based recombineering. For N-terminal GFP tagging of DYNC1H1, the N-FLAP tag was amplified from TH0478-R6Kamp-hNFLAP (Poser et al., 2008) using the forward and reverse GFP-tagging oligonucleotides (see Key Resources Table) and recombined onto the DYNC1H1 BAC via Redβγ mediated recombineering (Bird et al., 2011). The R1567E and K1610E mutations were then sequentially introduced into the CTD-2538J6 N-FLAP-DYNC1H1 BAC via counterselection based recombineering (Bird et al., 2011). For each mutation, the counterselection cassette (Bird et al., 2011) was amplified using the corresponding counterselection cassette oligonucleotides for R1567E and K1620E (see Key Resources Table) and recombined onto the DYNC1H1 BAC via Redβγ mediated recombineering. The counterselection cassette was then replaced by the appropriate rescue oligonucleotides for R1567E and 1610E (see Key Resources Table) via Redβ mediated recombineering. All BAC modifications were checked by PCR and sequencing. Integrity of the modified BACs was further assessed by digestion with restriction enzymes.

#### Generation of the BAC transgenic cell lines

BACs were purified using the NucleoBond Xtra BAC kit (Macherey Nagel) and transfected into HeLa Kyoto with the Effectene Transfection Reagent (QIAGEN) using the kit’s standard procedures. 24 hr after transfection, cells were trypsinised and diluted into passaging medium containing 300 μg/ml G418 (Serva) in order to select for single clones harboring the transfected BAC. Positive clones were screen for by Western Blot and Live cell imaging.

#### Cell lysis and immunoblotting

HeLa Kyoto and derivatives were lysed into a buffer containing 20mM Tris-HCl, pH 8.0, 100mM KCl, 1% Triton X-100 (Splinter et al., 2012), benzonase (25 U/mL) and a protease-inhibitors cocktail (Protease-inhibitor mix HP Plus, Serva). Lysates were diluted at 0.5 mg/ml into Laemmli buffer, boiled at 95°C and resolved on a 6% SDS-PAGE before transfer onto a nitrocellulose membrane. After blocking with 5% milk in PBS-Tween (0.1%), membranes were probed with antibodies diluted as follows: anti-alpha-tubulin DM1α, 1:5000 (Sigma-Aldrich); rabbit polyclonal anti-GFP, 1: 5000 (Abcam); mouse anti-p150^Glued^, 1:1000 (BD Transduction Laboratories); Sheep anti-mouse HRP and Donkey anti-rabbit HRP, 1:5000 (Amersham). All membranes were incubated with ECL reagent and developed onto Hyperfilm (Amersham) or imaged with the ChemiDoc™ MP Imaging System (BIO-RAD). The levels of images from scanned hyperfilm and ChemiDoc tiff-converted files were adjusted using FIJI and converted to 8-bit. Western blots against the dynein HC were performed as above with the following modifications; lysates were diluted to 0.75 - 1mg/ml in Laemmli buffer, blocking buffer was 5% BSA in PBS-Tween (0.1%), membranes were probed with Rat dynein HC antibody (Santa Cruz Biotechnology) at 1:250 dilution.

#### Immunofluorescence

For the nocodazole washout experiment, cells were seeded onto coverslips 24 hr before being synchronized with 10 μM RO-3306 (EMD-Millipore) for an extra 16 hr. Cells were then fixed with ice-cold methanol either before further treatment, after 30 min with 3.3 μM nocodazole, or after washout of the nocodazole for 3 min. After blocking for 1 hr with 5% BSA in PBS, coverslips were stained for 1 hr at 37°C with mouse anti-alpha-tubulin (Sigma-Aldrich), 1/400; goat anti-GFP (Hyman Lab, MPI-CBG Dresden), 1/5000; and rabbit anti-pericentrin (Abcam), 1/1000. Coverslips were washed three times with PBS and stained for 1 hr at 37°C with secondary antibodies: donkey anti-mouse DyLight 594 (Bethyl), 1/500; donkey anti-rabbit DyLight 650 (Bethyl), 1/300; and donkey anti-goat Alexa Fluor 488 (Invitrogen), 1/500.

For quantification of the mitotic phenotypes and mitotic localization of GFP-DHC and p150^Glued^, cells were seeded onto coverslips for 24 hr, and asynchronous cells were fixed using ice-cold methanol. After blocking for 1 hr with 5% BSA in PBS-Tween (0.1%), coverslips were stained for 1 hr at 37°C with rat anti-alpha-tubulin (Santa Cruz Biotechnology), 1/100; rabbit anti-GFP (Abcam), 1/500; and mouse anti- p150^Glued^ (BD Transduction Laboratories), 1/400. Coverslips were washed three times with PBS-Tween (0.1%) and stained for 1 hr at 37°C with secondary antibodies: donkey anti-rat DyLight 594 (Bethyl), 1/500; donkey anti-mouse Alexa Fluor 647 (Invitrogen), 1/300; and donkey anti-rabbit Alexa Fluor 488 (Invitrogen), 1/500.

All coverslips were mounted with ProLong Gold antifade reagent with DAPI (Invitrogen) and observed with a spinning-disk confocal system (Marianas, Intelligent Imaging Innovations) based on an Axio Observer Z1 microscope (ZEISS) equipped with an ORCA-Flash 4.0 camera (Hamamatsu Photonics). For all fixed cells quantification, images were taken as z sections of 0.2 μm using a 63 × 1.4 NA Apochromat objective (ZEISS).

Images illustrating common mitotic phenotype in HeLa transgenic lines were acquired using a DeltaVision imaging system (GE Healthcare) equipped with an sCMOS camera (PCO Edge 5.5) and using a 100 × 1.40 UPLS Apo objective (Olympus). Serial Z sections with 0.2 μm spacing were acquired and deconvolved using SoftWoRx software 6.1.l.

#### Live cell imaging with the ONIX system

48 hr before imaging, cells from a suspension at 2.10^6^ cells/ml were seeded in a CellASIC ONIX switching plate for mammalian cells (Merck Millipore). 16 hr before imaging, cells were synchronized with 10μM RO-3306 (EMD-Millipore) in CO_2_ independent media (GIBCO) supplemented with 10% FBS (GIBCO), 2mM L-Glutamine (PAN Biotech), Penicillin 100 U/ml and Streptomycin 0.1 mg/ml (PAN Biotech). Imaging was realized at 37°C with a DeltaVision imaging system (GE Healthcare) equipped with an sCMOS camera (PCO Edge 5.5) and using a 60 × 1.42 NA PlanApo-N objective (Olympus). For each time-point, 20 serial z sections of 0.75 μm with a binning of 4x4 were acquired. G2 synchronized cells were imaged before imaging media with 3.3 μM nocodazole and 10 μM RO-3306 was flowed into the CellASIC ONIX switching plate using a CellASIC ONIX Complete Perfusion System (Merck Millipore) with a flow rate of: 100 μL/hr. After 30 min nocodazole treatment, cells were imaged again before imaging media with 10 μM RO-3306 was flowed into the CellASIC ONIX switching plate with a flow rate of: 120 μl/hr. Upon nocodazole washout cells were imaged for 10 min.

### Quantification and Statistical Analysis

#### Ratio of phi- and open-dynein

In order to determine the ratio of phi and open-dynein in wild-type dynein (wtDyn) and mutant dynein (mtDyn) both samples were treated exactly the same during expression, purification and preparation of negative stain grids, as described above. Micrographs were randomly collected in different squares of the negative stain grids. Three rounds of 2D classification on each dataset were performed to remove bad particles. The high quality particles for both dynein datasets were then sub-classified into 100 sub-classes. In total, 4793 particles for wtDyn and 11819 particles for mtDyn were used in the classification and following quantification. Particles in these sub-classes were then assigned to one of 3 sub-datasets corresponding to either phi, open or ambiguous conformations. Ambiguous conformations refer to those particles that could not be easily assigned to phi or open conformations. Additional 2D classification on these sub-datasets was performed to make sure each sub-dataset only contains phi, open or ambiguous conformations. Any misassigned particles were moved to the correct sub-dataset. Manual inspection of the particles within each class confirmed that the different conformations had been grouped correctly. Averaged particle numbers of each class were calculated on 25 randomly selected micrographs for both wtDyn and mtDyn. After selection, the micrographs were removed from the dataset in order to avoid repetitive selection. This process was repeated 9 times and the mean and SEM were calculated.

#### Distance between motor domains

The distance between motor domains in wtDyn or mtDyn was directly measured from the 2D averages of negative stain data for open dynein. For DDB-bound dynein, dynactin density was subtracted from the images for focused 2D classification as described above. 90248 particles of wtDyn and 29733 particles for mtDyn were classified into 100 2D classes and 10086 DDB particles into 50 2D classes. The coordinates of the centers of each motor domain in each class were manually recorded. These values were then used to calculate the distance between the two motor domains in each class. The distance measurements were split into groups with 2.5nm interval each. The number of particles was summed for each distance group to estimate the distance distribution.

#### Motor domain orientation

In order to clearly determine the orientation of individual motor domains, small masks were generated according to the mass center of each motor domain in each class for local 2D refinement. This allowed us to obtain much better density of individual motor domains such that their orientations could be determined. Most motor domain particles were successfully assigned into two major classes (‘inverted’ or ‘parallel’) based on their stalk and linker orientations. Motor domains that could not be resolved after focused classification were defined as in an ambiguous orientation. Total particle numbers for each of the three major classes were summed to estimate the distribution of motor orientations in both open dynein (wtDyn and mtDyn) and DDB datasets. We confirmed that the distribution of motor orientations was similar in negative stain and vitreous ice.

#### Dynein microtubule affinity and processivity

All movie analysis was performed in FIJI (Schindelin et al., 2012). For kinetic analysis of dynein binding, 5-10 microtubules from each slide were selected at random. Kymographs along each microtubule were generated, and the number and duration of binding events greater than 2 frames (0.25 s) long was counted manually. For quantification of the microtubule binding rate and dwell time of single dynein complexes, 3 movies were collected (3 repeats over two different days) at two dynein concentrations (0.29nM and 0.58nM). The microtubule binding rate was calculated as the average number of binding events per second, per micrometer of microtubule, per nM dynein for each microtubule. Dwell time data was plotted as a histogram, with the bin size corresponding to one frame, and was fit to a two-phase exponential decay model (R^2^ > 0.99 for each fit). Dwell time was taken to be the fast-phase time constant of the fitted model (fast-phase was always in excess of 75% total weight. Each repeat was fit independently, and the six repeats each of wtDyn or mtDyn were averaged.

We used our microtubule binding rate to estimate K_on_ and the reciprocal of the dwell time constant to give K_off_. We estimated microtubule affinity (K_d_) for wtDyn and mtDyn by dividing the average K_off_ by average K_on_. This value was then multiplied by 802, corresponding to the number of dynein binding sites along 1 μm of microtubule. This figure was calculated assuming microtubules contain 13-protofilaments, with a lattice repeat of 8.1nm, and half of binding sites being inaccessible due to steric blocking by surface immobilization agents.

For DDB processivity measurements, 5-10 microtubules from 3 repeat movies (over 2 days) were used for kymograph analysis. The number of processive events (events lasting longer than 0.625 s and with unidirectional displacement greater than 500nm) per unit time (s), microtubule length (μm) and dynein amount (2.6nM) was measured. Data handling was performed in GraphPad Prism.

#### Quantification of gliding assay velocities

Gliding assay velocities were calculated by manually tracking the leading edge of moving microtubules using the FIJI plugin MTrackJ. The average velocity for the track of each microtubule was used to calculate the average velocity for the entire population of microtubules recorded.

#### Protein concentration and labeling efficiency

Protein concentrations were measured using Quick Start Bradford dye (Bio-Rad) and an Ultrospec 2100 Pro spectrophotometer (Amersham). Labeling efficiency of TMR-tagged dynein was measured using a Nanodrop ND1000 Spectrophotometer. Protein concentration was measured with A_280_ absorbance, and was used to calculate the dynein molarity using the extinction coefficient of 797340 M^-1^ cm^-1^, corresponding to a monomer of the full snap-tagged dynein complex. The ratio of TMR dye concentration (calculated directly by the spectrophotometer) to dynein protein concentration showed that the monomeric labeling efficiency was always at least 85%, with no difference observed between wtDyn and mtDyn. This corresponds to a labeling efficiency of at least 97.8% in the dimer.

#### Quantification of SEC traces

Analytical SEC was performed to separate the components formed when dynein, dynactin and BICD2N were mixed at a ratio of 1:2:20. To quantify the proportion of protein in the dynein-dynacin-BICD2N complex, fractions containing all components were identified by SDS-PAGE followed by SYPRO-Ruby staining as described above. The data from traces were plotted in GraphPad Prism and normalized to the baseline A280 reading at 5mL. The section of the trace corresponding to the DDB was extracted and the area under the curve (AUC) was calculated in GraphPad Prism. The DDB:total-protein ratio was calculated by dividing this by the AUC of the whole trace. This was repeated four times using three different preparations of the components. Statistical significance was determined by an unpaired, two-tailed t test.

#### Immunofluorescence data quantification

To quantify the GFP (Dynein) intensity at centrosomes, images of G2 cells with well-separated centrosomes (as visualized by PCNT staining) were acquired (at least 14 cells per condition and per experiment, N = 3 experiments, n > 87 centrosomes per condition). A SUM projection (16-bit in TIFF format) of the 11 slices surrounding each centrosome was created in FIJI and used for quantification. GFP fluorescence intensity at the centrosome was measured in a 12x12 pixels ROI centered on the centrosome (as visualized by PCNT staining). GFP cytoplasmic background was defined as the average fluorescence intensity measured from 3 ROIs (12x12 pixels) placed in the cytoplasm and subtracted from the GFP fluorescence intensity at centrosome.

To quantify the GFP-Dynein and p150^Glued^ intensities at spindle poles, images of whole metaphase cells were acquired (at least 19 cells per condition and per experiment, N = 3 experiments, n > 120 spindle poles per condition). A SUM projection (16-bit in TIFF format) of the 11 slices surrounding each spindle pole was created in FIJI, and used for quantification. GFP and p150^Glued^ fluorescence intensities were measured in a unique region of interest (ROI) of 12x12 pixels placed at the spindle pole, and were divided by the corresponding cytoplasmic background, as defined by the median fluorescence intensity from 3 ROIs (12x12 pixels) placed in the cytoplasm.

GFP and p150^Glued^ intensity data were analyzed using the R software. For each experiment, datasets were assessed for normality and homogeneity of the variances using a Shapiro-Wilk and a Bartlett tests, respectively. In all analyzed datasets, either normality or homogeneity of the variances was not reached and the datasets were analyzed using non-parametrical statistics. For a given variable, the presence of a difference between the different conditions was first assessed within each experiment using a Kruskall Wallis test. All conditions from an experiment were then further compared with paired Wilcoxon test. The displayed *p values* are the highest observed in 3 independent experiments.

#### Live cell data quantification

To quantify the GFP-DHC intensity recovery at microtubule dependent foci, 10 cells per condition were filmed with a 30 s time resolution over 10 min after nocodazole washout. For each time point, a MAX intensity z-projection (16-bit in TIFF format) was created using the hyperstack function of FIJI and used for quantification. The last time point of each movie (∼600 s) was used to center 3x3 pixels ROIs onto GFP foci that were identified as microtubule dependent (i.e., gradually appearing after nocodazole washout). The foci were tracked back in earlier time points and the corresponding ROIs were moved to follow the foci. When a focus disappeared the corresponding ROI was left unmoved for the previous time points. GFP fluorescence intensity at microtubule dependent foci was measured at every time point and the corresponding cytoplasmic background, as defined by the GFP fluorescence intensity from a 3x3 pixels ROI placed in the cytoplasm, was subtracted. For each time point, the average GFP intensity plus and minus standard deviation (SD) is displayed.

### Data and Software Availability

Six maps have been deposited with accession codes EMDB: EMD-3698, EMD-3703, EMD-3704, EMD-3705, EMD-3706, and EMD-3707. Atomic coordinates have been deposited with accession codes PDB: 5NUG, 5NVS, 5NVU, 5NW4, and 5NW4.

Cryo-EM data processing scripts and Gautomatch are available from K.Zhang’s website: http://www.mrc-lmb.cam.ac.uk/kzhang/. Scripts under development are available on request.

## Author Contributions

K.Z. performed EM analysis and structure determination. H.E.F. performed protein biochemistry, helped with EM data collection, and with S.E.L. performed single-molecule assays. A.R. and A.W.B. performed all cellular experiments. N.B.-B. advised on patient mutations. A.P.C. guided the project. H.E.F., A.P.C., A.W.B., A.R., K.Z., and S.E.L. prepared the manuscript.

## Supplemental Information

Document S1. Table S1

Movie S1. 2D Classification of Full-Length Phi-Dynein Reveals Its Flexibility, Related to Figure 1 Movie of phi-dynein 2D classes after sub-classification of cryo-EM images. The movie shows the flexibility of the tail with relation to the motor domains, as well as the conformational flexibility within the tail itself.

Movie S2. 3D Reconstruction and Architecture of Phi-Dynein, Related to Figure 1 The 15Å reconstruction of the whole dynein complex in the phi-particle form is shown first (grey, transparent). The 8.4Å map of the tail (cyan) and 3.8Å the map of the motor domains (purple) are fit into the 15Å map. Atomic models, generated by de novo modelling or fitting of previous str uctures 24 are shown inside the full map. NDD = N-terminal dimerization domain, HC = Heavy Chain, IC = Intermediate Chain, LIC = Light Intermediate Chain, MTBD = microtubule binding domain.

Movie S3. Conformational Changes between Two Distinct States of Phi-Dynein, Related to Figure 2 Two distinct conformations of the tail were observed after 3D classification: the ‘twisted’ form (purple) and the ‘parallel’ form (green). Each are initially displayed individually. The movie then shows the morphing between these two conformations (both colored purple). The major sites of change between the two states are around the N-terminus, the Robl light chain, and the density we assign to the LC8 and TcTex light chains.

Movie S4. Interaction between HCs in the Phi-Dynein Tail, Related to Figure 2 Electron density (mesh) and fitted models (HC A in light purple, HC B in dark purple, LIC A in light green, LIC B in dark green) show the third contact site in the dynein tail, between helical bundles 6 - 8 of the two HCs.

Movie S5. 2D Classes of Spontaneously Open wtDyn, Related to Figure 5 A selection of continuous 2D classes of spontaneously open wtDyn, in which the motor domains can be seen oriented with each stalk and neck pointing towards the center in the inverted conformat ion.

Movie S6. 2D Classes of Dynein in DDB, Related to Figure 5 A selection of continuous 2D classes of dynein in DDB with dynactin density subtracted from raw particles before classification. The necks and stalks emerge from the same side of the motor domains and point in the same direction so the motor orientation is parallel.

Movie S7. Conformational Changes between Phi-Dynein and Dynein in DDB, Related to Figure 7 Conformational changes in the dynein tail required to switch between phi -dynein and dynein in DDB. The 8.7Å DDB density and model are shown first, with main components labelled as in Figure S2. The structural model then morphs into the phi-dynein conformation, with the heavy chains adopting the twisted conformation around the N-terminal dimerization domain, together with associated changes in the heavy chain and accessory chains further down the tail. The model then merges back into the DDB conformation. The same transitions are then shown in the absence of electron density, followed by the same transitions shown in the context of the 12.4Å dynein tail map with a wider field of view. This shows the large-scale rotation of HC B in relation to HC A that occurs on dynactin binding.
