# Supplementary material for: Cryo-EM Reveals How Human Cytoplasmic Dynein Is Auto-inhibited and Activated
Source: Cell. 2017 Jun 15;169(7):1303–1314.e18. doi: 10.1016/j.cell.2017.05.025 (PMC5473941; doi:10.1016/j.cell.2017.05.025)
Supplement: Document S1. Table S1 [file mmc1.pdf]

Cell, Volume *169*

Supplemental Information

Cryo-EM Reveals How Human Cytoplasmic Dynein Is Auto-inhibited and Activated

Kai Zhang, Helen E. Foster, Arnaud Rondelet, Samuel E. Lacey, Nadia Bahi-Buisson, Alexander W. Bird, and Andrew P. Carter

Table S1. Cryo-EM data collection and processing summary, Related to Figure 1.

**Data Collection and processing**	**Dynein-(1)**	**Dynein-(2)**	**Dynein-(3)**	**DDB-(1)**	**DDB-(2)**
Institution	MRC-LMB	MRC-LMB	DLS	MRC-LMB	CEITEC
Detector	Falcon II	K2 summit	K2 summit	Falcon II	Falcon II
Frames	34	25	20	34	7
Exposure (sec)	2	20	8	2	2
Dose (ē/Å2)	54	40	40	54	54
Micrographs	6746	2649	3781	2634	3058
Pixel size (Å)	1.32	1.43	1.06	1.32	1.34
Raw particles	312, 198	187, 830	214, 543	75, 052	41, 222
Final particles	233,227 (motor) / 102,309 (tail)			78,671	
Resolution	3.8Å (motor) / 8.4Å (tail)			8.7Å / 12Å (masked tail)	

**Refinement summary**	
**Model composition of all proteins**	
Non-hydrogen atoms	46214
Amino acid residues	5852
Ligands (ADP/ATP)	6/2
**Refinement statistics**	
Resolution	257.28 - 3.80
sharpening B-factors (Å^2)	-100
Rfactor*	0.339
Overall FSC†	0.820
Correlation coefficient	0.922
Mean B-factor (Å^2)	157.1
**Rms deviations**	
Bond length (Å)	0.0060
Bond angle (°)	1.1182
Chiral volume (Å^3)	0.0699
**Validation**	
MolProbity score	2.13 (100th percentile)
Clashscore, all atom	19.17 (97th percentile)
Good rotamers	95.22%
**Ramachandran plot**	
Favored	94.87%
Outliers	0.10%
Cβ deviations >0.25Å	0.50%

*Rfactor = $(\Sigma||F_{obs}|-|F_{calc}||)/\Sigma|F_{obs}|$, in which $|F_{obs}|$ is the amplitude of simulated reflections from EM map and $|F_{calc}|$ is the amplitude of calculated reflections from the coordinates.

† Fourier Shell Correlation, $FSC_{overall} = \Sigma(N_{shell} FSC_{shell}) / \Sigma(N_{shell})$, where FSC_{shell} is the FSC in a given shell, N_{shell} is the number of 'structure factors' in the shell. $FSC_{shell} = \Sigma(F_{model} F_{EM})/(\sqrt{(\Sigma(|F|^2_{model}))}\sqrt{(\Sigma(|F|^2_{model}))})$.
