# Supplementary figures and images for: Cryo-EM Reveals How Human Cytoplasmic Dynein Is Auto-inhibited and Activated
Source: Cell. 2017 Jun 15;169(7):1303–1314.e18. doi: 10.1016/j.cell.2017.05.025 (PMC5473941; doi:10.1016/j.cell.2017.05.025)

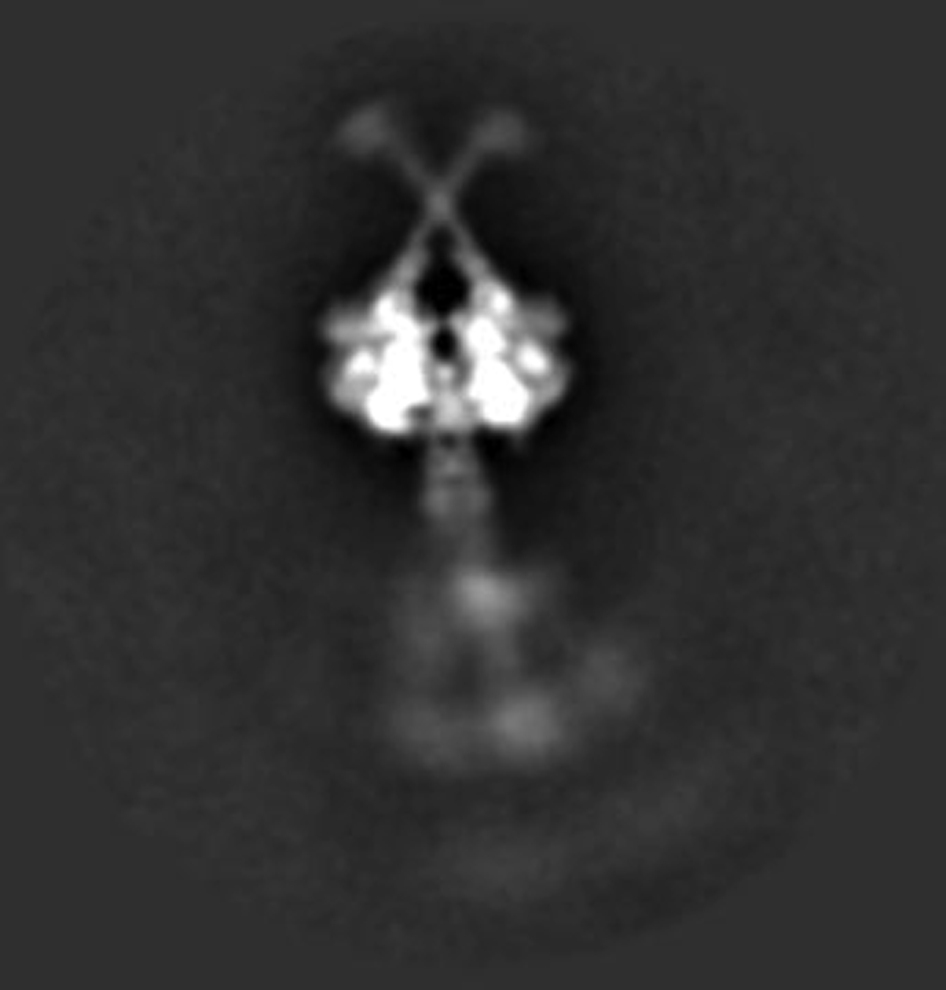

Supplement: Movie S1. 2D Classification of Full-Length Phi-Dynein Reveals Its Flexibility, Related to Figure 1 — Movie of phi-dynein 2D classes after sub-classification of cryo-EM images. The movie shows the flexibility of the tail with relation to the motor domains, as well as the conformational flexibility within the tail itself. [file mmc2.jpg]

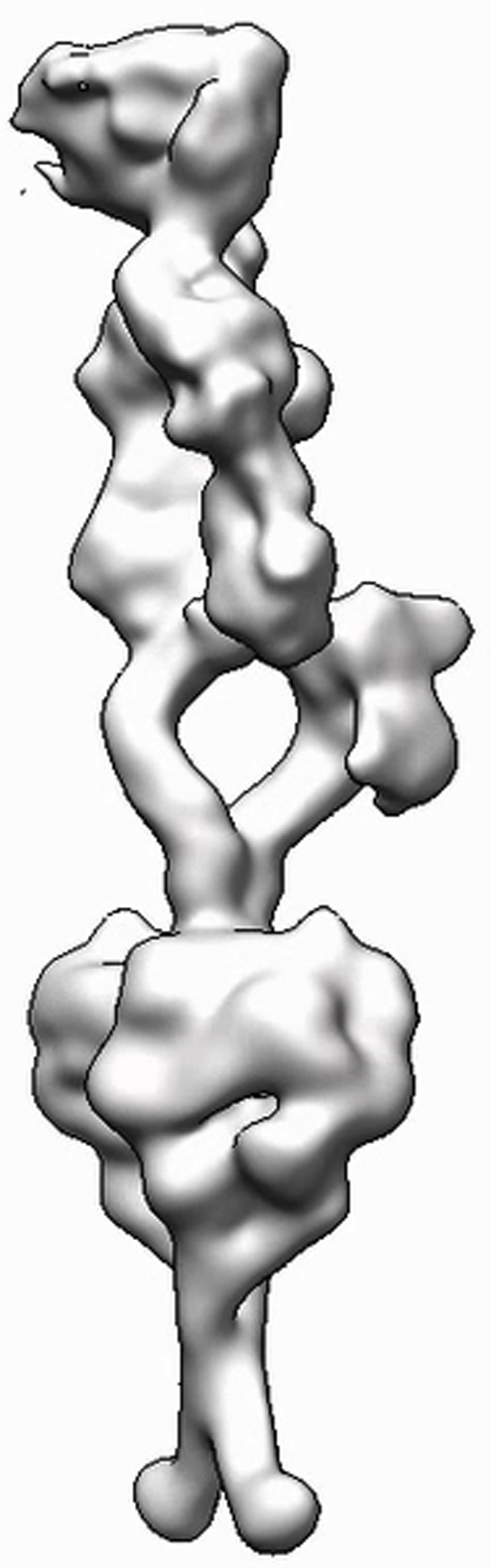

Supplement: Movie S2. 3D Reconstruction and Architecture of Phi-Dynein, Related to Figure 1 — The 15Å reconstruction of the whole dynein complex in the phi-particle form is shown first (grey, transparent). The 8.4Å map of the tail (cyan) and 3.8Å the map of the motor domains (purple) are fit into the 15Å map. Atomic models, generated by de novo modelling or fitting of previous str uctures 24 are shown inside the full map. NDD = N-terminal dimerization domain, HC = Heavy Chain, IC = Intermediate Chain, LIC = Light Intermediate Chain, MTBD = microtubule binding domain. [file mmc3.jpg]

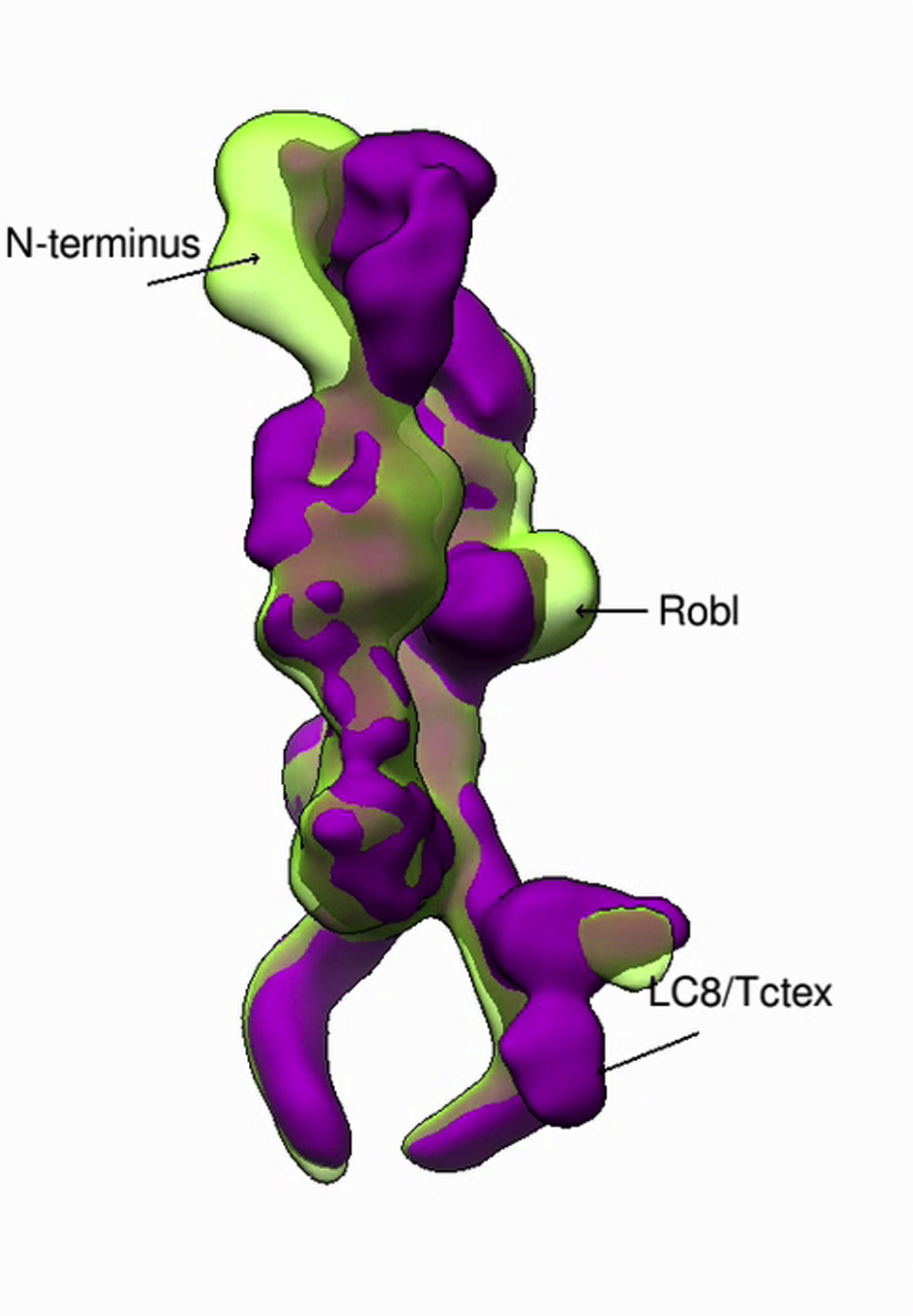

Supplement: Movie S3. Conformational Changes between Two Distinct States of Phi-Dynein, Related to Figure 2 — Two distinct conformations of the tail were observed after 3D classification: the ‘twisted’ form (purple) and the ‘parallel’ form (green). Each are initially displayed individually. The movie then shows the morphing between these two conformations (both colored purple). The major sites of change between the two states are around the N-terminus, the Robl light chain, and the density we assign to the LC8 and TcTex light chains. [file mmc4.jpg]

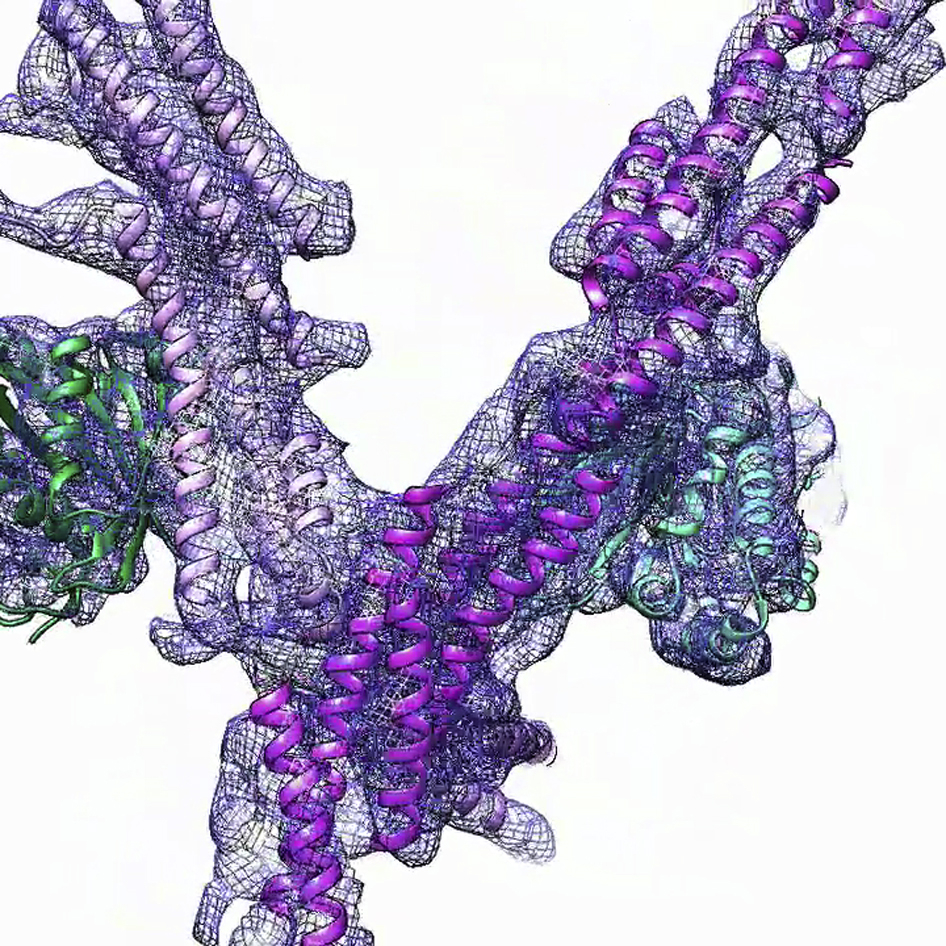

Supplement: Movie S4. Interaction between HCs in the Phi-Dynein Tail, Related to Figure 2 — Electron density (mesh) and fitted models (HC A in light purple, HC B in dark purple, LIC A in light green, LIC B in dark green) show the third contact site in the dynein tail, between helical bundles 6 - 8 of the two HCs. [file mmc5.jpg]

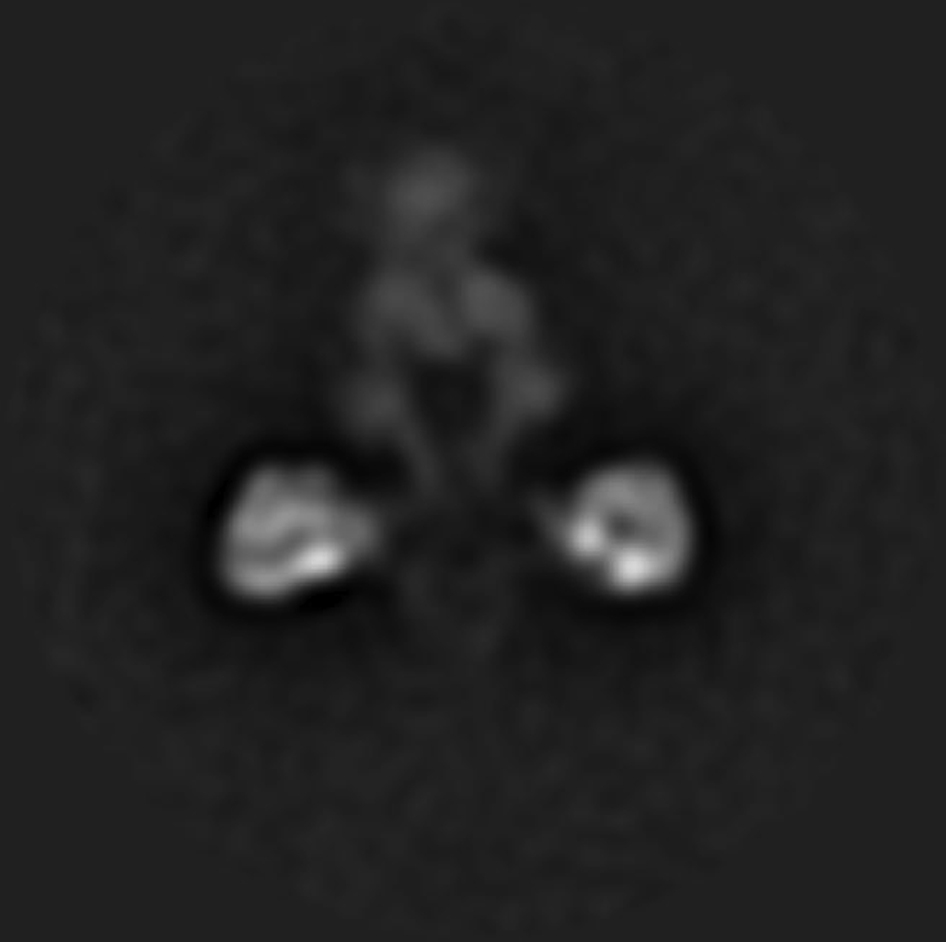

Supplement: Movie S5. 2D Classes of Spontaneously Open wtDyn, Related to Figure 5 — A selection of continuous 2D classes of spontaneously open wtDyn, in which the motor domains can be seen oriented with each stalk and neck pointing towards the center in the inverted conformat ion. [file mmc6.jpg]

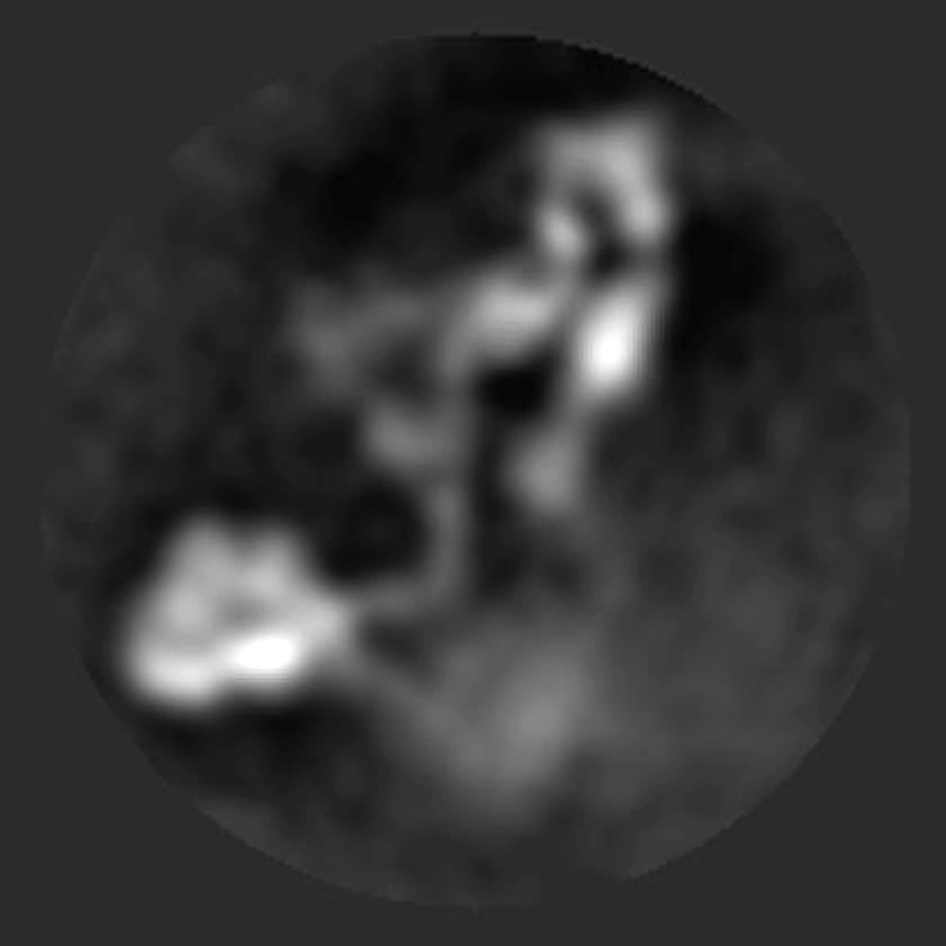

Supplement: Movie S6. 2D Classes of Dynein in DDB, Related to Figure 5 — A selection of continuous 2D classes of dynein in DDB with dynactin density subtracted from raw particles before classification. The necks and stalks emerge from the same side of the motor domains and point in the same direction so the motor orientation is parallel. [file mmc7.jpg]

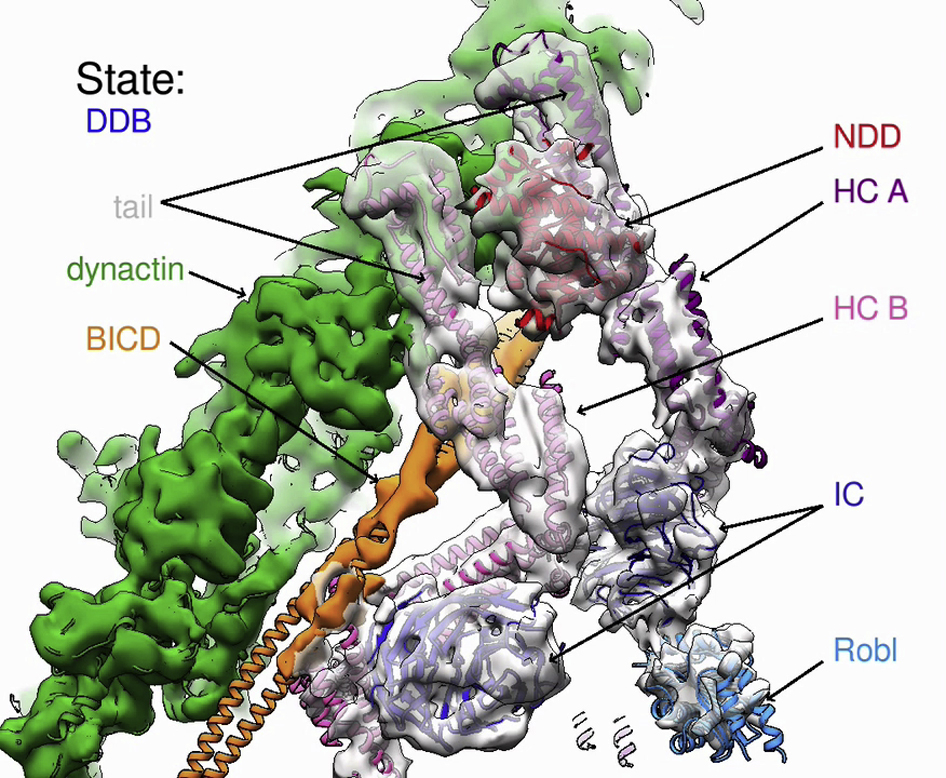

Supplement: Movie S7. Conformational Changes between Phi-Dynein and Dynein in DDB, Related to Figure 7 — Conformational changes in the dynein tail required to switch between phi -dynein and dynein in DDB. The 8.7Å DDB density and model are shown first, with main components labelled as in Figure S2. The structural model then morphs into the phi-dynein conformation, with the heavy chains adopting the twisted conformation around the N-terminal dimerization domain, together with associated changes in the heavy chain and accessory chains further down the tail. The model then merges back into the DDB conformation. The same transitions are then shown in the absence of electron density, followed by the same transitions shown in the context of the 12.4Å dynein tail map with a wider field of view. This shows the large-scale rotation of HC B in relation to HC A that occurs on dynactin binding. [file mmc8.jpg]
